# Altered zygotic gene expression caused by sperm with *Tdrd6* variants disrupts early embryonic development

**DOI:** 10.1002/mco2.70038

**Published:** 2025-01-05

**Authors:** Zhijie Hu, Yuqi Zhang, Renfei Cai, Qiuju Chen, Haiyan Guo, Danjun Li, Weidong Lin, Hongxi He, Haibo Wu, Yali Liu, Bin Li, Qianwen Xi, Hongyuan Gao, Jian Zhang, Qifeng Lyu, Yanping Kuang, Xuefeng Lu

**Affiliations:** ^1^ Department of Assisted Reproduction Shanghai Ninth People's Hospital Shanghai Jiao Tong University School of Medicine Shanghai China; ^2^ Medicinal Chemistry and Bioinformatics Center Shanghai Jiao Tong University School of Medicine Shanghai China

**Keywords:** artificial oocyte activation, assisted reproductive technology, early embryonic arrest, infertility, *TDRD6*

## Abstract

The precise mechanisms behind early embryonic arrest due to sperm‐related factors and the most effective strategies are not yet fully understood. Here, we present two cases of male infertility linked to novel *TDRD6* variants, associated with oligoasthenoteratozoospermia (OAT) and early embryonic arrest. To investigate the underlying mechanisms and promising therapeutic approaches, *Tdrd6* knock‐in and knock‐out mice were generated. The *Tdrd6* variant male mice demonstrated OAT and embryonic arrest, mirroring the clinical observations of our patients. Sperm from both affected individuals and mice exhibited aberrant localization of phospholipase C zeta and oocyte activation deficiency (OAD). The application of artificial oocyte activation (AOA) effectively overcame the infertility caused by the variants, facilitating successful pregnancies and live births in both human and mice. Additionally, our research revealed that OAD influences the expression of multitude of genes at the 2‐pronuclear (2PN) stage, with the *Mos* gene playing a pivotal role in early embryonic arrest. The injection of *Mos* mRNA can mitigate this arrest. To our knowledge, this is the first study to show that sperm‐related OAD affects gene expression at the 2PN stage and elucidate how AOA overcomes male factor‐derived embryonic arrest, enabling successful pregnancies and live births.

## Graphical abstract

*Tdrd6^−^^/−^* sperm have deficiency in intracellular Ca^2+^ oscillations and oocyte activation, which affects gene expression in the 2PN stage. The low expression of *Mos* in PN4 zygotes is the key to early embryonic arrest. AOA can rescue *Mos* gene expression, overcome male factor‐derived embryonic arrest, and achieve live births.

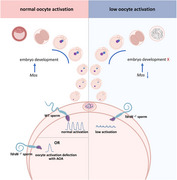

## 1 INTRODUCTION

Assisted reproductive technologies (ARTs) can help most infertile couples to conceive and give birth to their own biological offspring. However, the diagnosis and formulation of effective treatments for recurrent early embryonic arrest remain challenging for clinicians.^1^ Traditionally, it is believed that the factors affecting embryo development are exclusively maternally derived. Recently, evidence has suggested that semen delivers much more than just the paternal haploid genome to oocytes, and male factors are thought to be essential for appropriate embryonic development.^2^ Oocyte fertilization by a defective sperm cell was unable to survive past the first few sets of mitotic division.^3^ Paternal factors, such as the sperm centrioles, sperm proteins, sperm RNA, and epigenetic marks, can be transferred to the oocyte and may influence gene expression and normal early embryogenesis.^4^, ^5^, ^6^, ^7^ The important contributions of paternal factors to embryo development highlight the need for further research to understand the complex roles of male factors in early embryonic development to reveal approaches for enhancing and optimizing the effectiveness of ART.

In recent years, Tudor domain‐containing 6 (*TDRD6*) variants have been reported in patients with severe oligoasthenoteratozoospermia (OAT) and recurrent early embryonic arrest, indicating a causal relationship between *TDRD6* variants and failed ART.^8^, ^9^ TDRD6, which directly binds with PIWI, is a crucial component of chromatoid bodies (CBs), maintains the structural integrity of CBs, and facilitates spermiogenesis in mice.^10^ Because *TDRD6* is indispensable in *UPF1*‐mediated nonsense‐mediated mRNA decay (NMD),^11^ disruption of *TDRD6* may lead to abnormalities in the NMD pathway and may have incurable deleterious effects on early embryonic development in humans.^8^, ^9^

In this study, we report three novel variants of *TDRD6* in two men whose offspring encountered with early embryonic arrest. This type of observed infertility was confirmed to be of spermatic origin. We further investigated potential treatments by constructing a *Tdrd6* variant corresponding to the human variant knock‐in and *Tdrd6* knock‐out mouse models, leading to successful pregnancy and live birth in both humans and mice. Moreover, we revealed that embryonic development‐related gene expression in the 2‐pronuclear (2PN) stage is affected by oocyte activation deficiency, including effects on *Mos*, which persists from the oocyte stage to the zygote stage.

This study is the first to use a knock‐in mouse model to confirm that a biallelic *TDRD6* variant leads to OAT and sperm‐derived embryonic arrest. Furthermore, we found that an oocyte activation defect (OAD) is the major cause of early embryonic arrest in male infertility caused by the *TDRD6* variant and that intracytoplasmic sperm injection‐artificial oocyte activation (ICSI‐AOA) can be a successful therapeutic treatment for such cases in humans. Moreover, we revealed that OAD affects the expression of numerous genes in the 2PN stage and that *Mos* is the key gene affected during early embryonic arrest.

## 2 RESULTS

### 2.1 Identification of novel *TDRD6* variants

**FIGURE 1:**
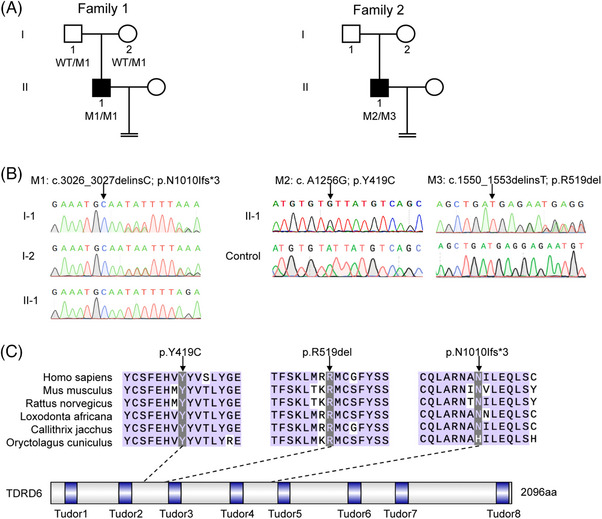
Identification of novel *TDRD6* variants. (A) Pedigree of two independent families affected by *TDRD6* variants. The black squares indicate the affected individuals. M, mutation; WT, wild type. (B) Sanger sequencing of *TDRD6* in subjects from family 1 and family 2. The black arrows indicate the locations of the variants. (C) Locations of *TDRD6* variants and conservation of the affected residues in six mammalian species. The black arrows indicate the locations of the variants.

According to whole exome sequencing (WES) analysis, we detected novel *TDRD6* variants in two infertile male individuals from two independent Chinese families with OAT and early embryonic arrest during ICSI treatment (Figure 1A). The affected individual in family 1 (II‐1) carried a homozygous frameshift variant, c.3026‐3027delinsC (p.N1010Ifs*3), which causes the protein to terminate prematurely, thus suggesting its strong deleterious effect. The affected individual in family 2 (II‐1) carried the compound heterozygous variants c.A1256G (p.Y419C) and c.1550‐1553delinsT (p.519del), which are potentially deleterious, as predicted by bioinformatic tools (Polyphen‐2, SIFT, and PROVEAN) (Table S1). All these variants were validated by Sanger sequencing (Figure 1B). The allelic frequencies of all three variants in the gnomAD, ExAC, and 1000 Genomes databases are listed in Table S1. *TDRD6* encodes a 2096‐amino acid protein containing eight Tudor domains, and the variants locations in the protein are shown in Figure 1C. Multiple sequence alignment indicated that the corresponding amino acid residues were highly conserved among different species (Figure 1C).

### 2.2 Clinical characteristics of affected individuals with *TDRD6* variants

Clinical characteristics of two affected male individuals with *TDRD6* variants who underwent ICSI are listed in Table 1. Patient II‐1 in family 1 is a 27‐year‐old male with 3‐year primary infertility and no underlying illnesses. In his ICSI attempt, his partner had 25 oocytes retrieved, 21 being metaphase II (MII) oocytes. Three 2PN zygotes were observed 16–18 h after ICSI, and 18 cleavage‐stage embryos were observed on day 3, 17 of which experienced embryonic arrest. Only one embryo developed into a blastocyst with a small cavity on day 5 and then into a 4CC‐grade blastocyst on day 6, which was ultimately discarded (Table 1). Patient II‐1 in family 2 is a 51‐year‐old male with 11‐year primary infertility and diabetes. He underwent an ICSI attempt at another center; 11 MII oocytes were retrieved from his partner, three of which were fertilized, but subsequent embryonic development was unclear. His 43‐year‐old partner did not become pregnant after the frozen embryo transfer (FET) cycle (Table 1). These clinical observations indicate a possible genetic contribution of *TDRD6* to early embryonic development.

**TABLE 1:** Clinical statistics of intracytoplasmic sperm injection (ICSI) attempts in affected individuals.

Patient	Age (year)	Infertility duration (year)	ICSI cycles								ICSI with AOA cycles							
			Insemination method	Total oocyte	MII oocyte	Fertilized outcomes (2PN + 1PN + MPN + 0PN)	Normal fertilization rate (%)	D3 Cleaved embryo	Embryo outcomes	Good‐quality embryo rate (%)	Insemination method	Total oocyte	MII oocyte	Fertilized outcomes (2PN + 1PN + MPN + 0PN)	Normal fertilization rate (%)	D3 Cleaved embryo	Embryo outcomes	Good‐quality embryo rate (%)
Family 1 II‐1	27	3	ICSI	25	21	3 + 1 + 0 + 17	3/21 (14.29)	18	17 arrested 1*4CC (D6)	0/18 (0.00)	ICSI + AOA	24	22	19 + 2 + 0 + 1	19/22 (86.36)	20	10 arrested 1*7CELL II 3*8CELL II 2*10CELL II 1*5CB (D5) 1*4CB (D5) 1*6BB (D5) 1*4CC (D5)	7/20 (35.0)
Family 2 II‐1	51	11	ICSI	11	11	Unknown 3 in total	Unknown	Unknown	Unknown	Unknown	ICSI + AOA	5	5	3 + 1 + 0	3/5 (60.0)	3	2 arrested 1*10CELL II	1/3 (33.33)
											ICSI + AOA	1	1	1 + 0 + 0	1/1 (100.0)	1	1*8CELL II‐	1/1 (100.0)

Abbreviations: AOA, artificial oocyte activation; MII, metaphase II; MPN, multipronucleus.

Both affected male individuals had abnormal semen parameters. Patient II‐1 in family 1 had a low sperm concentration, with B‐grade sperm extremely occasionally visible. Patient II‐1 in family 2 had a normal sperm concentration but low motility (Table S2). We investigated sperm morphology via hematoxylin and eosin (H&E) staining and observed that sperm from both patients exhibited severe sperm head malformation (Figure S1A). To further explore these sperm head abnormalities, we analyzed the ultrastructures of sperm from the affected individuals via transmission electron microscopy (TEM) and found that the deformed sperm head was characterized by a loosened perinuclear theca in which the acrosome was detached from the nuclear envelope, which was not observed in the sperm of healthy male controls (Figure S1B).

### 2.3 Deleterious *TDRD6* variants led to abnormal PLCζ distribution and defective oocyte activation

**FIGURE 2:**
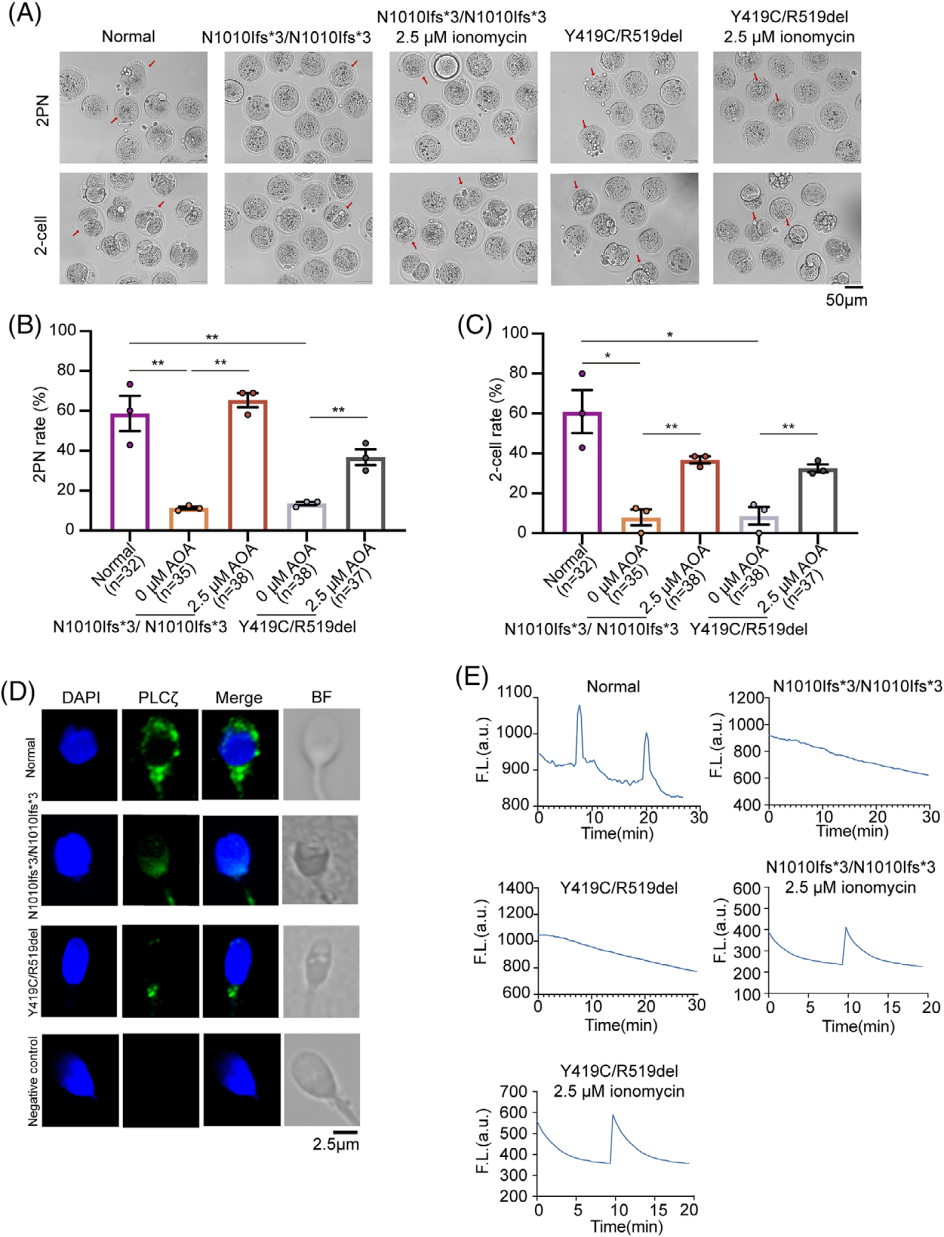
*TDRD6* variants caused abnormal phospholipase C zeta (PLCζ) distribution and failures to trigger Ca^2+^ oscillations, and intracytoplasmic sperm injection‐artificial oocyte activation (ICSI‐AOA) improved the oocyte‐activating capacity of sperm from patients with *TDRD6* variants. (A) Representative images of 2‐pronuclear (2PN) zygotes and two‐cell embryos generated by performing ICSI or ICSI‐AOA on wild‐type (WT) mouse oocytes using sperm from normal donors and patients with *TDRD6* variants. Scale bars, 50 µm. The red arrows indicate the representative zygotes and embryos in each embryonic stage. (B) Proportion of 2PN zygotes generated after performing ICSI or ICSI‐AOA on WT mouse oocytes using sperm from normal donors and patients with *TDRD6* variants. The bars indicate the mean ± SEMs. *p < 0.05, **p < 0.01, and ***p < 0.001. *n* = 3 biologically independent replicates per group. (C) Proportion of two‐cell embryos after performing ICSI or ICSI‐AOA on WT mouse oocytes using sperm from normal donors and patients with *TDRD6* variants. The bars indicate the mean ± SEMs. *p < 0.05, **p < 0.01, and ***p < 0.001. *n* = 3 biologically independent replicates per group. (D) Immunofluorescence staining for PLCζ (green) of sperm from normal donors and patients with *TDRD6* variants. 4',6‐diamidino‐2‐phenylindole (DAPI) (blue) was used to stain the pronucleus. BF, bright field. Scale bars, 2.5 µm. (E) Representative calcium oscillation patterns induced by sperm from normal donors and patients with *TDRD6* variants with and without 2.5 µM ionomycin AOA.

To investigate the oocyte‐activating capacity of sperm from patients with *TDRD6* variants, we carried out a mouse oocyte activation test (MOAT) in which oocytes from C57BL/6 female mice were injected with sperm from patients with *TDRD6* variants or normal sperm from healthy donors. The MOAT results clearly revealed that the percentages of 2PN and two‐cell embryos in the two affected individuals were significantly lower than those in the healthy donors, reflecting an oocyte‐activating capacity defect in the sperm from patients with *TDRD6* variants. The activating of mouse oocytes with 2.5 µM ionomycin after the injection of sperm from patients with *TDRD6* variants significantly increased the 2PN rate and two‐cell rate (Figure 2A–C, Figure S2). Phospholipase C zeta (PLCζ), a pivotal sperm‐borne oocyte activation factor located in the perinuclear theca, induces Ca^2+^ oscillations and initiates oocyte activation.^12^ We therefore explored PLCζ localization, PLCζ expression, and Ca^2+^ oscillation patterns in sperm from patients with *TDRD6* variants. Immunofluorescence revealed that PLCζ was located in the acrosomal region and neck of normally capacitated sperm; however, the PLCζ signal in sperm from patients with *TDRD6* variants was abnormally distributed, localizing to the neck alone and not to the acrosomal region (Figure 2D). Immunoblotting confirmed that PLCζ expression was lower in the sperm from patients with *TDRD6* variants than in that from normal donors (Figure S3). In addition, monitoring recorded 2 Ca^2+^ spikes within 30 min after injecting sperm from normal donors into mouse oocytes but no spikes when sperm from patients with *TDRD6* variants were used. However, activating mouse oocytes with 2.5 µM ionomycin after injection with sperm from patients with *TDRD6* variants resulted in a successful Ca^2+^ surge (Figure 2E). Taken together, these findings indicate that sperm from patients with *TDRD6* variants have defects in PLCζ distribution and expression that lead to a failure to evoke Ca^2+^ oscillations and consequent failure of oocyte activation.

Given that Tdrd6 interacts with Piwi family proteins, which play important roles in regulating gametogenesis,^10^ we investigated the potential functional changes in the p.N1010Ifs*3, p.Y419C, and p.519del variants by exploring whether *TDRD6* variants impaired the interaction of TDRD6 with PIWIL1. However, coimmunoprecipitation and immunoblotting assays demonstrated that none of the three variant sites affected the binding affinity between TDRD6 and PIWIL1 in HEK‐293T cells (Figure S4).

### 2.4 Male *Tdrd6* knock‐in and *Tdrd6* knock‐out mice presented poor fertility rates and early embryonic arrest after ICSI

**FIGURE 3:**
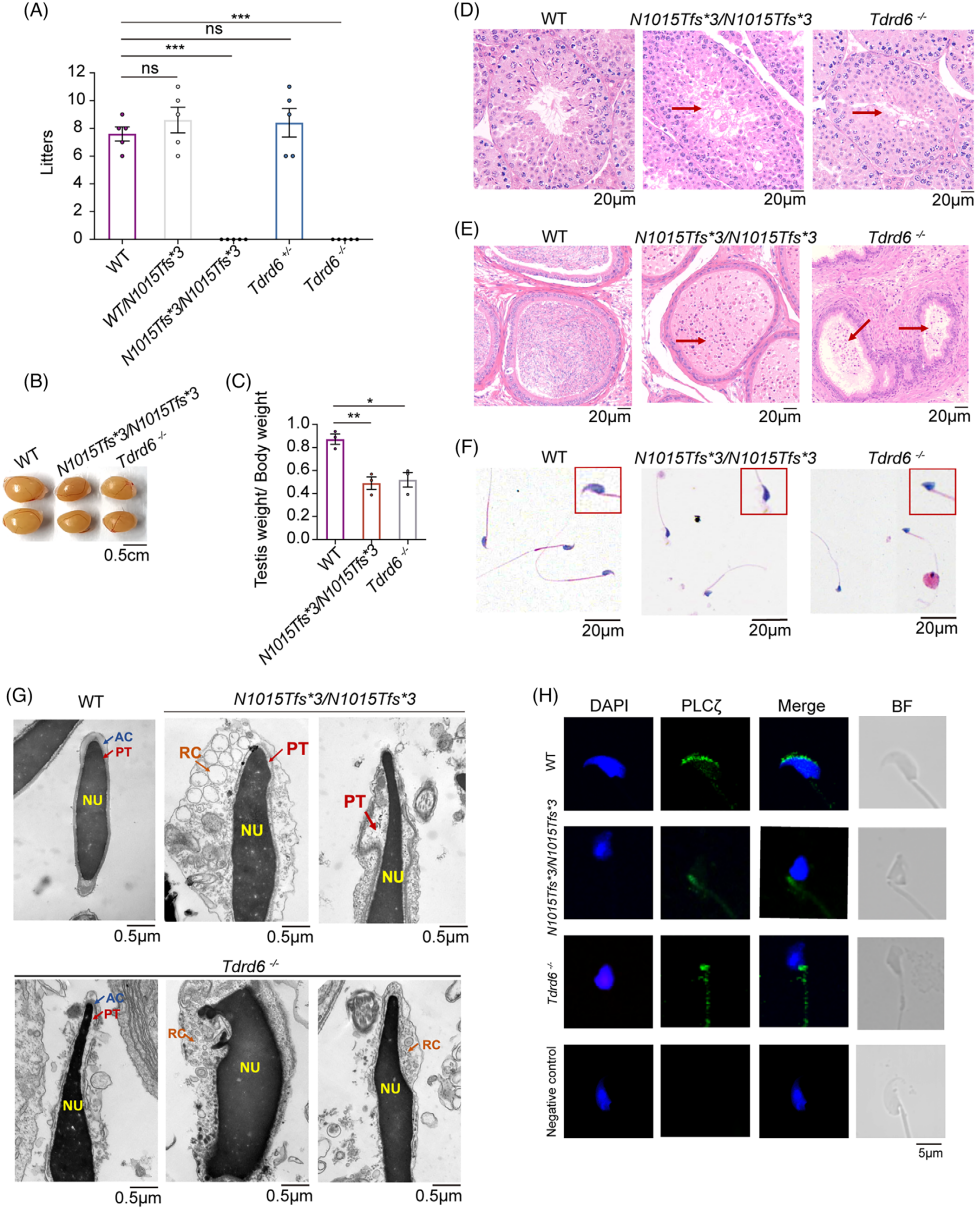
Tdrd6^N1015Tfs*3/N1015Tfs*3^ and *Tdrd6^−/−^* male mice presented with sperm head malformation and abnormal phospholipase C zeta (PLCζ) distribution. (A) Male wild‐type (WT), Tdrd6^N1015Tfs*3/N1015Tfs*3^, and *Tdrd6^−/−^* mice were mated with WT females. The circles indicate the number of pups born to each female mouse. The bars indicate the mean ± SEMs. *p < 0.05, **p < 0.01, and ***p < 0.001. (B) Testes from 10‐week‐old WT, Tdrd6^N1015Tfs*3/N1015Tfs*3^, and *Tdrd6^−/−^* male mice. Scale bars, 0.5 cm. (C) Quantification of the testis weight/body weight ratio in WT, Tdrd6^N1015Tfs*3/N1015Tfs*3^, and *Tdrd6^−/−^* male mice. The bars indicate the mean ± SEMs. *n* = 3 biologically independent mice per group. (D) Hematoxylin‐eosin (HE) staining of testes from WT, Tdrd6^N1015Tfs*3/N1015Tfs*3^, and *Tdrd6^−/−^* male mice. Scale bars, 20 µm. The red arrows indicate that the elongated spermatids were lacking in the testes. (E) HE staining of epididymides from WT, Tdrd6^N1015Tfs*3/N1015Tfs*3^, and *Tdrd6^−/−^* male mice. Scale bars, 20 µm. The red arrows indicate that the elongated spermatids were lacking in the epididymides. (F) HE staining of sperm from WT, Tdrd6^N1015Tfs*3/N1015Tfs*3^, and *Tdrd6^−/−^* male mice. The magnified panel with the red box shows views of the sperm heads. Scale bars, 20 µm. (G) Transmission electron microscopy images of the ultrastructures of sperm from male WT, Tdrd6^N1015Tfs*3/N1015Tfs*3^, and *Tdrd6^−/−^* mice. AC, acrosome; PT, perinuclear theca; NU, nucleus; RC, residual cytoplasm. Scale bars, 0.5 µm. (H) Immunofluorescence staining for PLCζ (green) in sperm from WT, Tdrd6^N1015Tfs*3/N1015Tfs*3^, and *Tdrd6^−/−^* male mice. DAPI (blue) was used to stain the pronucleus. Scale bars, 5 µm.

To further explore the functional changes in p.N1010Ifs*3, we generated *Tdrd6* knock‐in (Tdrd6^N1015Tfs*3/N1015Tfs*3^) mice harboring the deleterious variant c.3026‐3027delinsC (p.N1010Ifs*3) and *Tdrd6* knock‐out (*Tdrd6^−/−^*) mice via the CRISPR‐Cas9 system (Figure S5A,B). The male Tdrd6^N1015Tfs*3/N1015Tfs*3^ mice were infertile, similar to *Tdrd6^−/−^* mice (Figure 3A). Similar to *Tdrd6^−/−^* mice, Tdrd6^N1015Tfs*3/N1015Tfs*3^ males presented with smaller testes (Figure 3B) and lower testis weight/body weight ratios than wild‐type (WT) males did at 10 weeks of age (Figure 3C). Histological analysis of the testes and epididymis revealed that sperm were nearly absent from the epididymides of Tdrd6^N1015Tfs*3/N1015Tfs*3^ mice and *Tdrd6^−/−^* mice and that elongated spermatids were lacking, suggesting disrupted spermatogenesis (Figure 3D,E). According to H&E staining (Figure 3F) and TEM analysis, compared with those of WT mice, the sperm of Tdrd6^N1015Tfs*3/N1015Tfs*3^ and *Tdrd6^−/−^* mice displayed irregular sperm head shapes, as well as abnormal nuclear forms, excess residual cytoplasm, acrosome detachment, and a loosened perinuclear theca structure (Figure 3G). These results showed that similar to *Tdrd6^−/−^* mice, Tdrd6^N1015Tfs*3/N1015Tfs*3^ male mice suffered severe spermatogenesis defects, leading to malformation of the sperm head and infertility.

A previous study revealed that *Tdrd6* maintains the structural integrity of the CB, we used TEM to examine CB formation in Tdrd6^N1015Tfs*3/N1015Tfs*3^ and *Tdrd6^−/−^* male mice. We observed that CB was near the nuclear membrane in round spermatids across all groups. CB morphology was fully condensed in WT male mice; in contrast, CB in Tdrd6^N1015Tfs*3/N1015Tfs*3^ and *Tdrd6^−/−^* male mice was diffuse and less condensed (Figure S6), indicating that CB formation was disrupted by *Tdrd6* deficiency, which is consistent with previous findings.

We then examined the PLCζ location and expression in Tdrd6^N1015Tfs*3/N1015Tfs*3^ and *Tdrd6^−/−^* mice. Immunofluorescence revealed a PLCζ signal in the acrosomal region of WT sperm but in the necks of sperm in the Tdrd6^N1015Tfs*3/N1015Tfs*3^ mice, which is similar to the findings for the *Tdrd6^−/−^* mice (Figure 3H). Immunoblotting revealed that PLCζ expression was lower in the sperm of Tdrd6^N1015Tfs*3/N1015Tfs*3^ and *Tdrd6^−/−^* mice than in that of WT mice (Figure S7). Thus, the Tdrd6^N1015Tfs*3/N1015Tfs*3^ and *Tdrd6^−/−^* male mice presented abnormal PLCζ distributions and low PLCζ expression in sperm.

**FIGURE 4:**
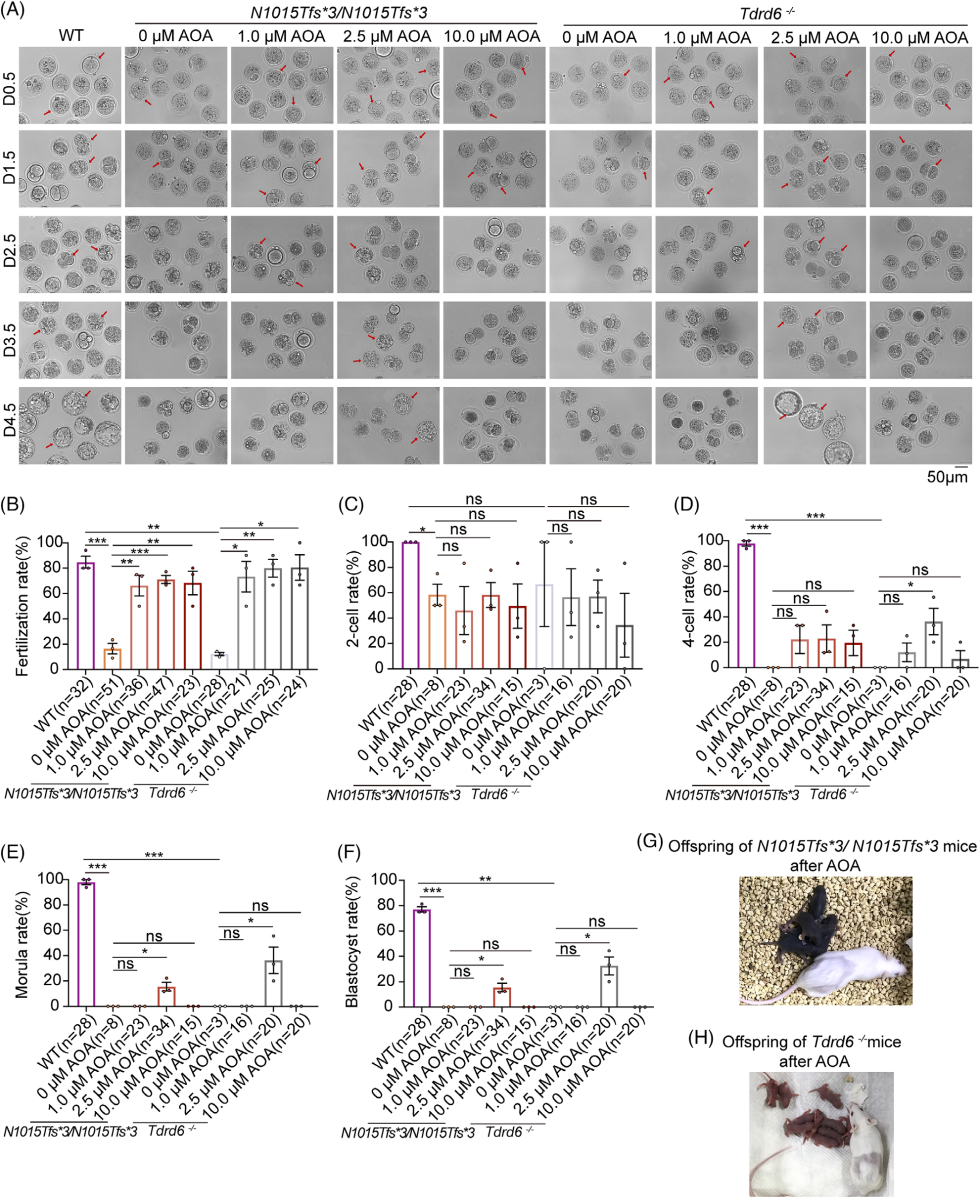
Intracytoplasmic sperm injection‐artificial oocyte activation (ICSI‐AOA) with 2.5 µM ionomycin overcame male infertility in Tdrd6^N1015Tfs*3/N1015Tfs*3^ and *Tdrd6^−/−^* mice. (A) Representative images of embryonic development after ICSI or ICSI‐AOA with different concentrations of ionomycin using the sperm of wild‐type (WT), Tdrd6^N1015Tfs*3/N1015Tfs*3^, and *Tdrd6^−/−^* mice. Scale bars, 50 µm. *n* = 3 biologically independent male mice in each group. The red arrows indicate the representative 2‐pronuclear (2PN) zygotes on D0.5, two‐cell embryos on D1.5, four‐cell embryos on D2.5, morulas on D3.5, and blastocysts on D4.5, respectively. (B–F) Fertilization rate, two‐cell rate, four‐cell rate, morula rate, and blastocyst rate after ICSI or ICSI‐AOA with different concentrations of ionomycin. The bars indicate the mean ± SEMs. *p < 0.05, **p < 0.01, and ***p < 0.001. (G) Representative images of the offspring of Tdrd6^N1015Tfs*3/N1015Tfs*3^ male mice born after ICSI‐AOA with 2.5 µM ionomycin. (H) Representative images of the offspring of *Tdrd6^−/−^* male mice born after ICSI‐AOA with 2.5 µM ionomycin.

We performed ICSI using sperm from Tdrd6^N1015Tfs*3/N1015Tfs*3^ and *Tdrd6^−/−^* mice to examine their oocyte activation ability and embryonic development. Compared with those of WT sperm, the fertilization rates of sperm from the Tdrd6^N1015Tfs*3/N1015Tfs*3^ and *Tdrd6^−/−^* mice were significantly lower, and the embryos eventually arrested at an early stage accompanied by fragmentation and failure to develop into morulas and blastocysts (Figure 4A–F, Figure S8).

Taken together, sperm from the Tdrd6^N1015Tfs*3/N1015Tfs*3^ and *Tdrd6^−/−^* mice presented abnormal PLCζ distributions, and the Tdrd6^N1015Tfs*3/N1015Tfs*3^ and *Tdrd6^−/−^* male mice presented poor fertility rate and early embryonic arrest after ICSI, which is consistent with observations from human patients.

### 2.5 Rescue of the *Tdrd6/TDRD6* defect in ICSI with proper AOA in mice and humans

As a chemical oocyte‐activating agent, ionomycin is widely used to overcome fertilization failure, but its effectiveness in overcoming early embryonic arrest is unclear. Therefore, we performed ICSI‐AOA with different working concentrations of ionomycin (1.0, 2.5, and 10.0 µM) on Tdrd6^N1015Tfs*3/N1015Tfs*3^ and *Tdrd6^−/−^* mice to investigate whether it could improve fertilization and embryonic development. Surprisingly, compared with non‐AOA treatment, treatment with 1.0, 2.5, or 10.0 µM ionomycin significantly increased the fertilization rates of the sperm from Tdrd6^N1015Tfs*3/N1015Tfs*3^ and *Tdrd6^−/−^* mice. However, only ICSI‐AOA with 2.5 µM ionomycin successfully overcame the early embryonic arrest for the sperm from the Tdrd6^N1015Tfs*3/N1015Tfs*3^ and *Tdrd6^−/−^* mice and significantly improved the blastocyst rate; the embryos activated by 1.0 µM and 10.0 µM ionomycin were unable to develop into morulas or blastocysts (Figure 4A–F, Figure S8). To further investigate whether Tdrd6^N1015Tfs*3/N1015Tfs*3^ and *Tdrd6^−/−^* mice can produce their own offspring via ICSI‐AOA with 2.5 µM ionomycin, two‐cell embryos were transplanted into pseudopregnant mice. Encouragingly, the embryos successfully implanted and developed into normal pups (Figure 4G,H).

**FIGURE 5:**
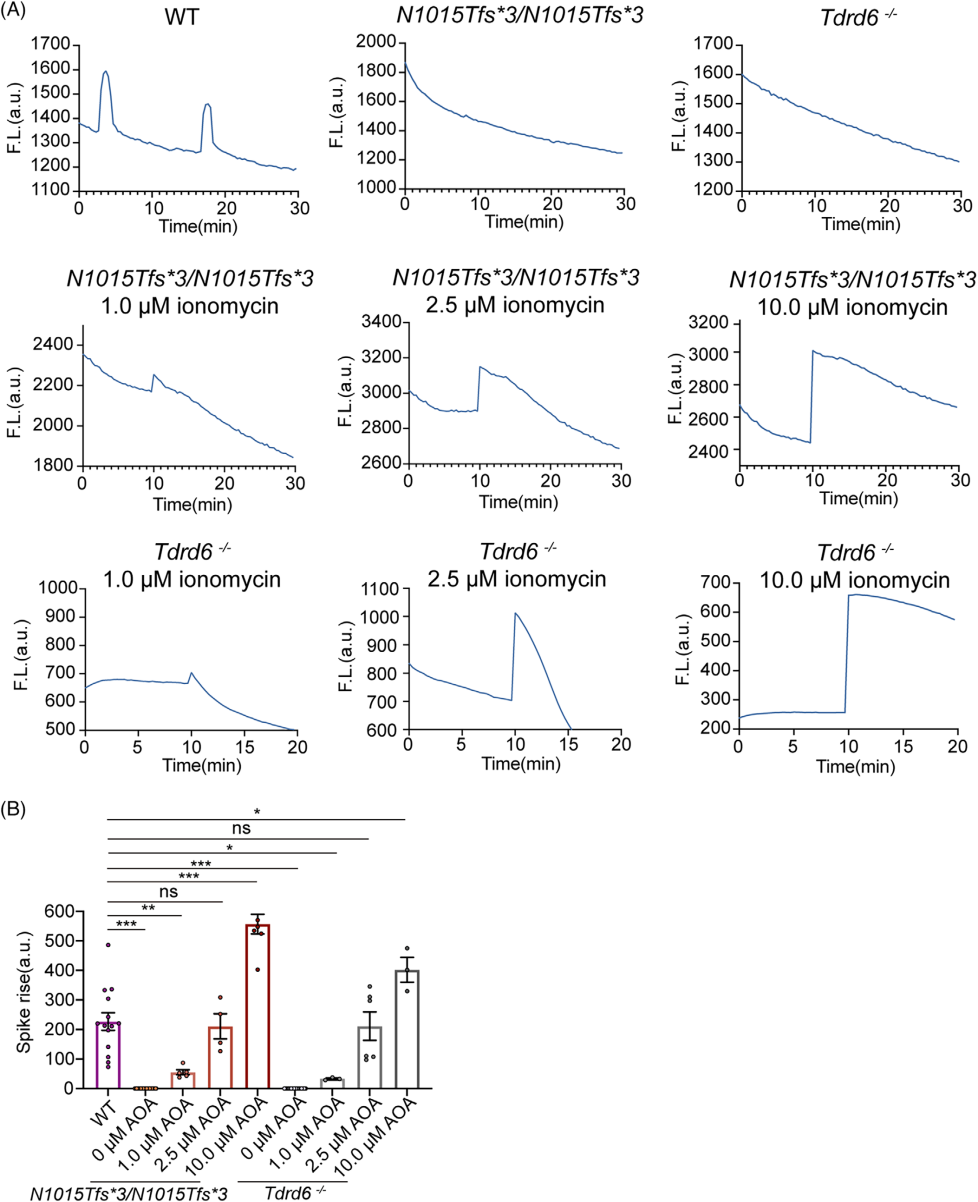
Calcium oscillation patterns of Tdrd6^N1015Tfs*3/N1015Tfs*3^ and *Tdrd6^−/−^* sperm after intracytoplasmic sperm injection (ICSI) or intracytoplasmic sperm injection‐artificial oocyte activation (ICSI‐AOA). (A) Representative calcium signal graphs for Tdrd6^N1015Tfs*3/N1015Tfs*3^ and *Tdrd6^−/−^* sperm after ICSI or ICSI‐AOA using different concentrations of ionomycin. (B) Amplitude of the Ca^2+^ surge elicited by Tdrd6^N1015Tfs*3/N1015Tfs*3^ and *Tdrd6^−/−^* sperm after ICSI or ICSI‐AOA with different concentrations of ionomycin. The bars indicate the mean ± SEMs. *p < 0.05, **p < 0.01, and ***p < 0.001.

We further analyzed the Ca^2+^ oscillation patterns of the WT, Tdrd6^N1015Tfs*3/N1015Tfs*3^, *Tdrd6^−/−^*, and AOA with 1.0, 2.5, and 10.0 µM ionomycin groups. We observed 2 Ca^2+^ spikes within 30 min after ICSI with WT sperm but no spikes when the sperm from the Tdrd6^N1015Tfs*3/N1015Tfs*3^ and *Tdrd6^−/−^* mice were used (Figure 5A). The Ca^2+^ oscillation graph demonstrated that ionomycin resulted in a single Ca^2+^ surge, but it was unable to elicit normal Ca^2+^ oscillation. During the single Ca^2+^ surge, we observed a fast increase followed by a gradual decrease (Figure 5A). In particular, a comparison of the amplitudes of the Ca^2+^ surge at different concentrations of ionomycin indicated that the amplitude of the calcium signal tended to increase as the ionomycin concentration increased. Compared with that in the WT group, the amplitude of the Ca^2+^ surge elicited by 2.5 µM ionomycin was comparable to that evoked by WT sperm; however, the amplitude elicited by 1.0 µM ionomycin was significantly lower and that elicited by 10.0 µM ionomycin was significantly greater (Figure 5A,B). On the basis of these experimental results, we speculate that 1.0 µM ionomycin induced an inadequate Ca^2+^ surge and that 10.0 µM ionomycin induced an excessive Ca^2+^ surge, neither of which would enable appropriate embryonic development. However, 2.5 µM ionomycin induced a near‐physiological calcium amplitude, significantly improving embryonic development and rescuing OAD and early embryonic arrest raised by the sperm in Tdrd6^N1015Tfs*3/N1015Tfs*3^ and *Tdrd6^−/−^* mice.

To explore whether ICSI‐AOA is a therapeutic treatment for male infertility in humans, we performed ICSI‐AOA with 10.0 µM ionomycin in two affected male individuals. Patient II‐1 in family 1 successfully achieved a 35% good‐quality embryo rate, with fewer early arrested embryos than in the ICSI‐alone cycle (Table 1). In his first FET cycle, we transferred two embryos (eight CELL II and 10 CELL II) on day 3 and achieved an intrauterine singleton pregnancy culminating in the birth of a healthy baby girl; her physical and psychomotor development was normal, no malformations were diagnosed after birth, and no special medical or surgical treatments or hospital visits were recorded. In a second, more recent FET cycle, we transferred two embryos (seven CELL II and eight CELL II) from this patient on day 3, and his wife achieved an intrauterine singleton pregnancy, which is currently ongoing. Patient II‐1 in family 2 attained good‐quality embryo rates of 33.3% and 100% in two ICSI‐AOA treatment cycles (Table 1), but no embryos implanted after FET. His wife was 42 years old when this procedure was performed, which might be partially responsible for the implantation failure. Here, we demonstrated that proper AOA is an effective therapeutic treatment for male infertility caused by *TDRD6* variants in humans and can enhance oocyte activation ability, improve embryo quality, and lead to the conception of a healthy baby.

### 2.6 Abnormal expression of *Mos* in zygotes causes early embryonic arrest

**FIGURE 6:**
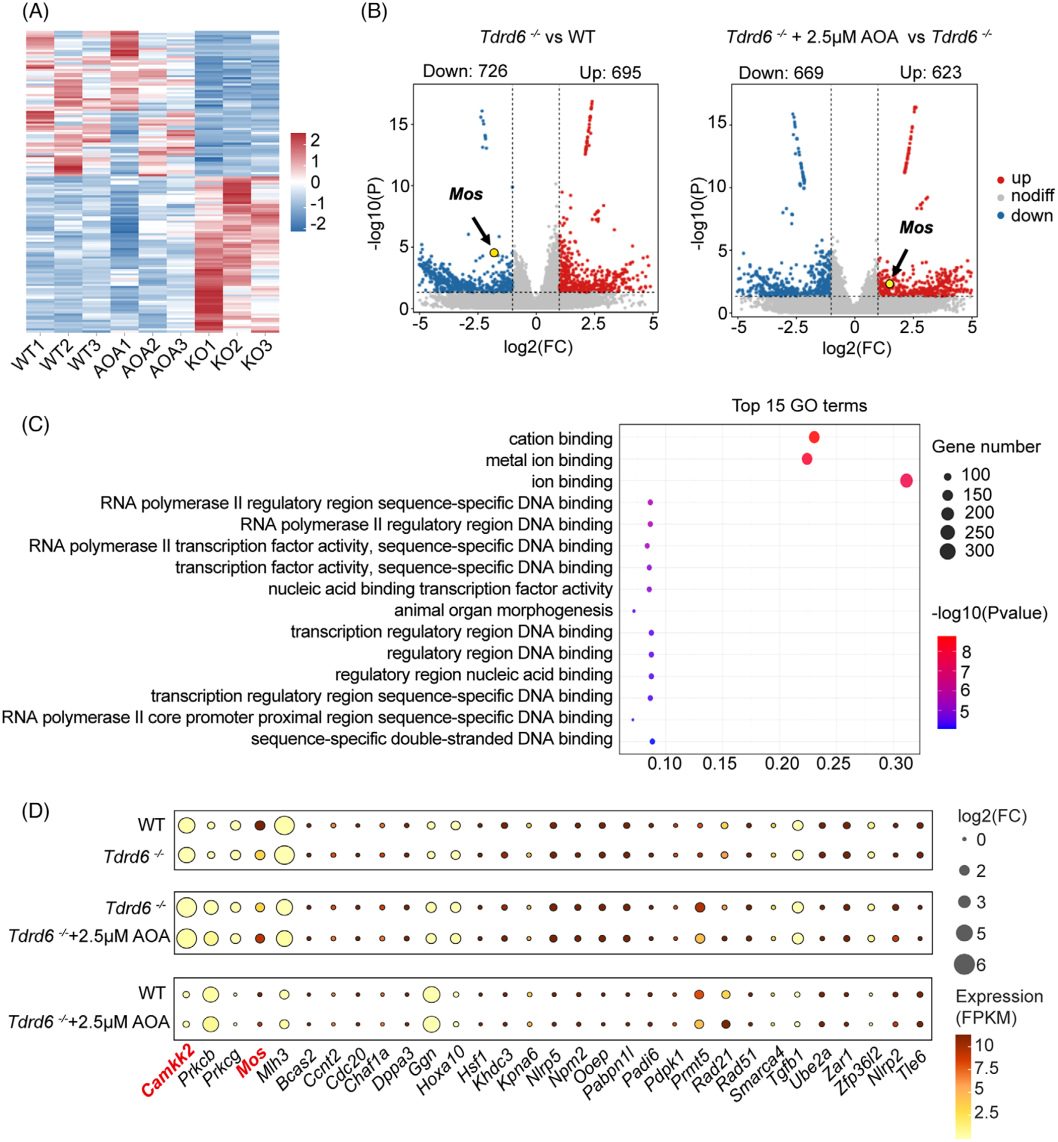
RNA‐seq analysis of the transcriptomes of the zygotes of WT, *Tdrd6^−/−^*, and *Tdrd6^−/−^*‐ICSI‐AOA groups. (A) Heatmap analysis of the wild‐type (WT) group, *Tdrd6^−/−^* group, and *Tdrd6^−/−^*‐ICSI‐AOA group. Three biological replicates were performed for each group, and for each replicate, six 2‐pronuclear (2PN) zygotes were collected 6 h after intracytoplasmic sperm injection (ICSI). (B) Volcano map analysis of the WT group, *Tdrd6^−/−^* group and *Tdrd6^−/−^*‐ICSI‐AOA group, with arrows indicating the *Mos* gene. (C) Gene Ontology (GO) functional analysis of the genes with significant differences in expression observed between the WT group and *Tdrd6^−/−^* group and between the *Tdrd6^−/−^* group and *Tdrd6^−/−^*‐ICSI‐AOA group, and no significant differences in expression between the WT group and the *Tdrd6^−/−^*‐ICSI‐AOA group. (D) Bubble map showing the expression of genes related to embryonic development for the WT group, *Tdrd6^−/−^* group, and *Tdrd6^−/−^*‐ICSI‐AOA group. The color and size of the dots indicate the expression level (FPKM) and the absolute value of the log2‐transformed fold change, respectively. *Camkk2* was noted for its large fold change. *Mos* was noted for its high expression level and large fold change.

To explore the mechanisms through which ionomycin AOA overcomes the early embryonic arrest in *Tdrd6^−/−^* mice, we conducted RNA sequencing on zygotes from the WT group (ICSI with WT sperm), *Tdrd6^−/−^* group (ICSI with *Tdrd6^−/−^* sperm), and *Tdrd6^−/−^*‐ICSI‐AOA group (ICSI with *Tdrd6^−/−^* sperm, activated by 2.5 µM ionomycin). The gene expression levels of three replicates within each group were strongly correlated (Figure 6A). We identified 726 downregulated transcripts and 695 upregulated transcripts in zygotes from *Tdrd6^−/−^* group compared with WT group and 669 downregulated transcripts and 623 upregulated transcripts in zygotes from *Tdrd6^−/−^*‐ICSI‐AOA group compared with *Tdrd6^−/−^* group (Figure 6B). We performed Kyoto Encyclopedia of Genes and Genomes (KEGG) and Gene Ontology (GO) analyses on a total of 1196 genes whose expression significantly differed between WT group and *Tdrd6^−/−^* group, significantly differed between *Tdrd6^−/−^* group and *Tdrd6^−/−^*‐ICSI‐AOA group, but did not significantly differ between WT group and *Tdrd6^−/−^*‐ICSI‐AOA group (Figure 6C, Figure S9). GO analysis revealed that these genes were involved mainly in cation binding (256 genes), metal ion binding (249 genes), ion binding (346 genes), etc. (Figure 6C), indicating the essential role of Ca^2+^ regulation in oocyte activation. KEGG analysis revealed that these genes were enriched in 44 subcategories in six KEGG classifications (Figure S9). Among these 1196 genes, 105 genes were enriched in signal transduction. Volcano map and bubble map analyses revealed several genes related to the calcium signaling pathway (*Camkk2*, etc.) and embryonic development (*Mos*, *Mlh3*, etc.) (Figure 6D). Thus, we revealed that OAD involves changes in gene expression in 2PN zygotes. Moreover, qPCR confirmed *Mos* and *Camkk2* were significantly downregulated in *Tdrd6^−/−^* group (compared with WT group) and significantly upregulated in *Tdrd6^−/−^*‐ICSI‐AOA group (compared with *Tdrd6^−/−^* group), reaching levels comparable to those in WT group.

*Camkk2* encodes calmodulin‐dependent protein kinase kinase 2; the mRNA expression of *Camkk2* persists until the zygote and two‐cell stages, but its role in embryo development is unclear.^13^ RNA sequencing revealed large fold changes in *Camkk2* expression between the WT group and the *Tdrd6^−/−^* group and between the *Tdrd6^−/−^* group and the *Tdrd6^−/−^*‐ICSI‐AOA group. In addition, our RNA sequencing data revealed high‐level expression of *Mos* and large fold changes in *Mos*, which previous studies have indicated is a crucial gene related to early embryonic arrest.^14^, ^15^ Therefore, we hypothesized that the low‐level expression of *Camkk2* or *Mos* might be a potential mechanism of early embryonic arrest in *Tdrd6^−/−^* mice.

**FIGURE 7:**
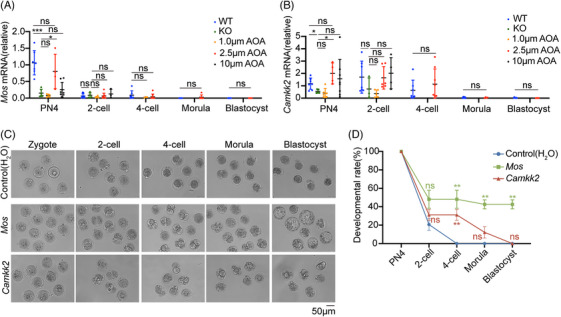
Fertilization with *Tdrd6^−/−^* sperm causes abnormal expression of *Mos* in zygotes and consequent early embryonic arrest. (A) qRT‐PCR results showing the relative expression of *Mos* at different stages of embryonic development after intracytoplasmic sperm injection (ICSI) with sperm from wild‐type (WT) or *Tdrd6^−/−^* mice or after intracytoplasmic sperm injection‐artificial oocyte activation (ICSI‐AOA) with different concentrations of ionomycin. Each circle represents a separate embryo. The bars indicate the mean ± SEMs. *n* = 3 biologically independent male mice in each group. (B) qRT‐PCR was used to determine the relative expression of *Camkk2* during different stages of embryonic development after ICSI with sperm from WT or *Tdrd6^−/−^* mice or after ICSI‐AOA with different concentrations of ionomycin. Each circle represents a separate embryo. The bars indicate the mean ± SEMs. *n* = 3 biologically independent male mice in each group. (C) Representative images of embryonic development at different stages after *Tdrd6^−/−^* zygotes were injected with H_2_O (control), *Mos*, or *Camkk2* mRNA. Scale bars, 50 µm. (D) Quantification of embryonic development at different stages after *Tdrd6^−/−^* zygotes were injected with H_2_O (control), *Mos*, or *Camkk2* mRNA. The bars indicate the mean ± SEMs. ^*^ *p* < 0.05, ^**^ *p* < 0.01, and ^***^ *p* < 0.001. *n* = 3 biologically independent male mice in each group.

To confirm the expression levels of *Mos* and *Camkk2* in embryos, we performed qRT‐PCR at different embryonic developmental stages in WT zygotes and *Tdrd6^−/−^* zygotes treated with ionomycin at different concentrations. qRT‐PCR revealed that at PN4, the *Mos* expression level was significantly lower in the *Tdrd6^−/−^* group than the WT group. The *Mos* expression levels in the 1.0 µM AOA group and 10.0 µM AOA group were comparable to the *Tdrd6^−/−^* group, and the *Mos* expression level in the 2.5 µM AOA group was significantly greater than the *Tdrd6^−/−^* group and comparable to the WT group. *Mos* expression was almost undetectable at the two‐cell stage onwards (Figure 7A). The qRT‐PCR results revealed that at PN4, the expression level of *Camkk2* was significantly lower in the *Tdrd6^−/−^* group than the WT group. The *Camkk2* expression levels in the 1.0 µM AOA group and 10.0 µM AOA group were comparable to the *Tdrd6^−/−^* group, and the *Camkk2* expression level in the 2.5 µM AOA group was significantly greater than that in the *Tdrd6^−/−^* group and comparable to that in the WT group. *Camkk2* expression at two‐cell stage was comparable among all groups and almost undetectable at later developmental stages (Figure 7B). These results demonstrated that the expression levels of *Mos* and *Camkk2* were indeed low at PN4 stage in *Tdrd6^−/−^* mice and that treatment with 2.5 µM AOA rescued *Mos* and *Camkk2* expression in *Tdrd6^−/−^* PN4‐staged embryos.

We subsequently microinjected *Tdrd6^−/−^* PN4 zygotes with mouse *Mos* or *Camkk2* mRNA and observed the ensuing embryonic development. Compared with the controls, zygotes microinjected with *Camkk2* mRNA presented an increased percentage of four‐cell embryos, but the embryos did not develop into blastocysts. In contrast, microinjection of *Mos* mRNA successfully overcame early embryonic arrest and significantly increased the blastocyst rate (Figure 7C,D), suggesting that *Mos* plays an important role in *Tdrd6^−/−^* zygote development.

To explore the precise mechanism of the low‐level expression of *Mos* mRNA in PN4 zygotes in the *Tdrd6^−/−^* group, we examined the degradation of *Mos* transcript in PN4 zygotes through the microinjection of mRNA mix, including GFP with the *Mos* 3′‐UTR, mCherry with the *Tubb3* 3′‐UTR (unaffected gene), and BFP with polyA (Figure S10A). We found that the GFP and mCherry fluorescence intensities in PN4 zygotes were comparable between the WT group and the *Tdrd6^−/−^* group (Figure S10B–D), suggesting that *Tdrd6* defects did not influence the degradation of *Mos* transcript in PN4 zygotes.

We further investigated *Mos* mRNA expression during fertilization and cleavage in WT zygotes. The qRT‐PCR results revealed that *Mos* mRNA was highly expressed in WT MII oocytes and decreased in expression level after fertilization. The expression level of *Mos* mRNA remained low during the PN1 to PN3 stages, increased slightly during the PN4 to PN5 stages, and then continued to decrease during the presyngamy period and subsequent development (Figure S11).

The *Mos* expression level in the 10.0 µM AOA group was comparable to that in the *Tdrd6^−/−^* group, and previous studies have shown that elevated reactive oxygen species (ROS) levels can affect gene expression during early embryonic development.^16^ We speculated that AOA with 10.0 µM ionomycin produces excessive ROS, inhibiting *Mos* mRNA expression. We found that the ROS fluorescence intensity in the 10.0 µM AOA group was significantly higher than that in the WT group, and the addition of vitamin C (VitC) to the culture medium (10.0 µM AOA + VitC group) significantly decreased the ROS level compared with that in the 10.0 µM AOA group (Figure S12A,B). Furthermore, the qRT‐PCR results revealed that the *Mos* mRNA expression level was significantly higher in the 10.0 µM AOA + VitC group than in the 10.0 µM AOA group (Figure S12C). These results demonstrated AOA with 10.0 µM ionomycin produces excessive ROS, inhibiting *Mos* mRNA expression, which can be ameliorated by the addition of VitC.

The biological mechanism through which *Mos* participates in zygote and early embryonic development is still unclear. Taken together, these results indicate that *Tdrd6* defects lead to decreased *Mos* expression in PN4 zygotes, ultimately resulting in early embryonic arrest and male infertility in *Tdrd6^−/−^* mice.

## 3 DISCUSSION

Biallelic *TDRD6* variants have been previously reported in male infertility patients with severe OAT and sperm‐derived embryonic arrest.^8^, ^9^ However, no variants have been confirmed, and the role of *TDRD6* in early embryonic development is still unclear. In this report, we described two human cases of OAT involving variants in *TDRD6* and demonstrated that *Tdrd6* nullizygosity in mice causes OAT, as well as early embryonic arrest. By constructing a mouse model with a *Tdrd6* knock‐in variant, we confirmed that a homozygous missense variant in *Tdrd6* led to OAT and early embryonic arrest.

Further study revealed that sperm from men and mice with *TDRD6/Tdrd6* variants presented abnormal localization of PLCζ and OAD. AOA could successfully overcome such sperm‐derived infertility and result in live births in humans and mice. These data provide robust evidence that the loss of *TDRD6* causes OAT and ICSI treatment failure and that proper AOA can overcome embryonic arrest and achieve live births for affected couples. The age of the female partner is the main cause of ART failure. In this study, the wife of patient 2 was 43 years old when the ART cycle combined with AOA was performed at our center and failed to become pregnant after embryo transfer. The age‐related decline in oocyte quality may be responsible for the failure of embryo implantation in this patient. Notably, the daughter of the patient with the *TDRD6* variant in family I was born healthy, and no special medical or surgical treatments or hospital visits were recorded in the follow‐up monitoring after 3 years. There were also no observed malformations in the offspring of the Tdrd6^N1015Tfs*3/N1015Tfs*3^ and *Tdrd6^−/−^* mice. These observations indicated that disruption of the *TDRD6* gene does not influence subsequent embryonic or offspring development.

Here, we noticed that patient II‐1 in family 2 had a normal sperm concentration but low sperm motility and severe sperm head malformation. His sperm concentration is contrary to previous reports, which revealed that deficiency of *Tdrd6* disrupted the differentiation of spermatozoa from round spermatids to elongated spermatid and led to a significant decrease in sperm counts.^17^ The various phenotypic effects could be attributed to the varying degrees of functional impairment in *TDRD6* caused by distinct variations, as demonstrated in other genes.^18^, ^19^, ^20^ A recent report also revealed that patients with *TDRD6* variants had a wide range of variation in the sperm concentration, with one of five subjects exhibiting the total sperm count close to the normal, indicating significant individual differences.^8^ Therefore, we speculated that the more pronounced phenotypic effects exhibited by patient II‐1 in family 1 are due to the functions of *TDRD6* being affected by the variation. However, this speculation needs to be further verified.

TDRD6 interacts with PIWI and Mvh and is essential to the CB structure and for proper maturation and precursor miRNA expression.^10^ A previous study revealed embryonic arrest and supposed that abnormalities in the NMD pathway might exert deleterious effects on early embryonic development.^9^ Here, we revealed an abnormal distribution of PLCζ in sperm and a lack of [Ca^2+^]_i_ oscillations after ICSI with the sperm from men and mice with *TDRD6/Tdrd6* variants. AOA can increase intracellular calcium levels and overcome embryonic arrest. The transfer of embryos developed after AOA can result in healthy offspring. These results indicate that disrupted PLCζ expression and localization in sperm from men and mice with *TDRD6/Tdrd6* variants leads to early embryonic arrest.

Understanding the exact mechanisms of paternal contribution may assist reproductive scientists and clinicians in determining new causes of early embryonic arrest.^2^, ^7^ AOA is a promising method for addressing fertilization issues during in vitro fertilization and embryo transfer cycles. The use of calcium ionophores and other stimulators can induce oocyte activation and potentially lead to successful fertilization. Recently, two studies showed that AOA treatment could be a potential treatment method for male factor‐derived embryonic arrest.^21^, ^22^ However, the specific role that oocyte activation plays in regulating the early stages of embryonic development has yet to be determined. In this study, we found that insufficient [Ca^2+^]_i_ oscillations after ICSI contribute to gene expression changes in 2PN zygotes (PN4), including changes in *Mos* expression, leading to early embryonic arrest. The appropriate AOA can rectify *Mos* expression deficiencies caused by insufficient oocyte activation. Moreover, injection of *Mos* mRNA in 2PN zygotes (PN4) generated with *Tdrd6^−/−^* sperm is sufficient to prevent early embryonic arrest. Our finding revealed a paternal effect on early embryonic development through the regulation of *Mos* at the 2PN stage (PN4). The *Mos* gene encodes a serine/threonine protein kinase and is expressed predominantly in oocytes. *Mos* is instrumental in triggering the MAPK signaling cascade, which is essential for oocyte meiotic division.^23^, ^24^ Recent clinical research has indicated that women with biallelic mutations in the *MOS* gene experience infertility due to early embryonic arrest.^15^, ^25^ Consistent with these results, we found that the injection of *Mos* mRNA can prevent the embryonic arrest caused by *Tdrd6* deficiency. These results demonstrated that the regulation of *Mos* expression by oocyte activation at the 2PN stage is a potential driving factor for subsequent embryonic development. Interestingly, only AOA with 2.5 µM ionomycin, not AOA with higher concentration (10.0 µM) of ionomycin, could promote *Mos* gene expression and prevent early embryonic arrest.

To investigate the precise regulatory mechanisms of *Mos* mRNA levels underlying early embryonic arrest due to oocyte activation, mRNA stability was first analyzed during early embryonic development. Our results demonstrated that the decrease in *Mos* expression in *Tdrd6^−/−^*‐derived zygotes after fertilization is not related to *Mos* mRNA degradation. Moreover, we noted that the *Mos* mRNA level was upregulated at the PN4 stage during normal embryonic development. Therefore, we speculate that the variation in the expression of the *Mos* gene may be due to the influence of gene expression regulation. Previous studies have shown that elevated ROS levels can affect gene expression during early embryonic development.^16^ To determine whether the decrease in *Mos* expression is due to excessive ionomycin activation, we detected *Mos* expression after antioxidant treatment. Our results revealed that *Mos* expression was lower in the corresponding control group (10.0 µM ionomycin) than in the group with additional VitC intervention (10.0 µM ionomycin + 25 µg/mL VitC). Therefore, high concentration of ionomycin (10.0 µM) may lead to increased ROS production, which in turn inhibits *Mos* expression. These results indicated that the concentration of calcium, ionomycin, and other stimulators might affect the treatment outcome of AOA, and care should be taken when AOA is performed to prevent male factor‐derived early embryonic arrest.

Our study constructed a model for *Tdrd6* variants that lead to early embryonic developmental arrest. The conclusions related to the regulatory mechanisms involving *Mos* found in this model cannot be directly generalized to other patients with early embryonic arrest caused by male factors. Mutations in *TDRD6* may result in different, specific expression patterns during early embryonic development. This limits the applicability of our research. Further work is needed to investigate the molecular mechanisms of the dose‐dependent effects and whether *Mos* gene expression regulation by [Ca^2+^]_i_ oscillations is a common mechanism in embryo development.

## 4 MATERIALS AND METHODS

### 4.1 Study participants

We recruited 37 couples with infertility due to early embryonic arrest from the Department of Assisted Reproduction at Shanghai Ninth People's Hospital. All participants in the study provided written informed consent. The study was approved by the Shanghai Ninth People's Hospital Ethics Committee (SH9H‐2022‐T328‐1). All animal studies were approved by the Laboratory Animal Ethics Committee of the Ninth People's Hospital Affiliated with Shanghai Jiao Tong University (SH9H‐2023‐A796‐1).

### 4.2 ICSI and AOA in mice

After mouse MII oocytes were collected, the MII oocytes were recovered in KSOM embryo medium (MR‐101‐D, Millipore) for 1 h in an incubator (37°C, 5% CO_2_), after which ICSI was performed. Sperm were obtained from 10‐week‐old C57BL/6 male mice via the swim‐up method. The sperm head was injected into the oocyte, avoiding the spindle, as previously described.^26^ The embryos were cultured in KSOM in an incubator (37°C, 5% CO_2_). For AOA, after 30 min of ICSI, the oocytes were continuously activated by exposure to ionomycin (I0634, Sigma) for 10 min. The working concentrations of ionomycin were 1.0, 2.5, and 10.0 µM. Then, the oocytes were fully washed and cultured in KSOM in an incubator (37°C, 5% CO_2_) to observe fertilization and embryonic development. Pronucleus formation was observed 6 h after ICSI.

### 4.3 ICSI and AOA for infertile individuals with *TDRD6* variants

Oocyte retrieval was carried out 32–36 h after hCG induction. Single sperm was injected into MII oocytes under a microscope equipped with a manipulator (Narishige, Japan), and then they were exposed to 10.0 µM ionomycin (I0634, Sigma) (diluted with G‐IVF medium) for 10 min. Then, the oocytes were completely washed with G‐IVF medium and cultured in sequential media (G1/G2) in an incubator. Pronucleus formation was observed 16–18 h after ICSI (day 1), cleavage was observed on day 3, and blastocyst formation was observed on day 5 or 6. The embryos were graded according to Cummins standards.^27^ The normal fertilization rate was defined as the ratio of the number of 2PN zygotes to the number of MII oocytes injected. A good‐quality embryo was defined as a cleavage‐stage embryo of grade I or II on day 3 or a blastocyst of grade ≥3BB on day 5 or 6.

### 4.4 Mouse oocyte activation test

MOAT was performed as previously described.^28^, ^29^, ^30^, ^31^ In brief, sperm from each patient were injected into mouse MII oocytes, while sperm from donors with proven fertility served as a positive control. The two‐cell formation rate at 30‐h postinjection (the number of two‐cell embryos divided by the number of surviving injected oocytes) was used to calculate the sperm activation ability.

### 4.5 RNA sequencing (RNA‐seq)

Each sample contained six zygotes that were collected after 6 h post‐ICSI (PN4‐5). Pronuclear morphology and hours postinsemination were considered when classifying the pronuclear stage according to previous studies.^32^, ^33^ RNA extraction was carried out via a total RNA microextraction kit (Zymo Research), RNA‐seq libraries were constructed via a TruSeq RNA sample preparation kit (Illumina), and sequencing was performed via an Illumina NovaSeq 6000 instrument from Genergy Biotechnology Co., Ltd. The raw data were processed via Skewer (v0.2.2), and the data quality was assessed via FastQC v0.11.2. The sequencing reads had a length of 2 × 150 bp. The gene expression level was calculated as fragments per kilobase of transcript per million mapped reads. DESeq2 was used to screen differentially expressed genes among the different groups (*p* < 0.05 and |log2‐fold change| ≥1). GO term and KEGG pathway enrichment analyses were conducted via DAVID Bioinformatics Resources and OmicShare CloudTools, respectively.

### 4.6 In vitro transcription and microinjection of zygotes

To prepare mRNAs for microinjection in the *Mos* and *Camkk2* rescue experiments, total RNA was extracted from fresh C57BL/6 mouse brains via a FastPure Cell/Tissue Total RNA Isolation Kit (RC112, Vazyme Biotech) and then reverse transcribed into cDNA as a template for subsequent PCR via a HiScript III 1st Strand cDNA Synthesis Kit (R312, Vazyme Biotech). Specific primers were designed to PCR amplify the coding sequences of *Mos* and *Camkk2* via PCR and to append a T7 promoter (Table S3). In vitro transcription of *Mos* and *Camkk2* was performed with a HiScribe T7 ARCA mRNA Kit (E2060S, New England Biolabs). A MEGAclear Transcription Clean‐Up Kit (AM1908, Invitrogen) was used for mRNA purification. *Mos* mRNA, *Camkk2* mRNA (50 ng/µL in RNase‐free water), or H_2_O was injected into zygotes 6 h post‐ICSI (PN4‐5). Microinjection was performed with a FemtoJet injector (Eppendorf). After injection, the zygotes were cultured in KSOM in an incubator (37°C, 5% CO_2_).

### 4.7 Statistical analysis

At least three replicates were carried out for each experiment. GraphPad Prism 9.3.1 software and Statistical Package for Social Sciences version 26.0 were used for statistical analysis. The data are presented as the mean ± SEM. *p* < 0.05 was considered to indicate statistical significance.

## AUTHOR CONTRIBUTIONS

Xuefeng Lu, Yanping Kuang, Qifeng Lyu, and Jian Zhang designed the study and reviewed the manuscript. Zhijie Hu and Yuqi Zhang performed the experiments, analyzed the data, and drafted the article. Renfei Cai, Qiuju Chen, Haiyan Guo, and Danjun Li collected the data and revised the article. Renfei Cai and Qiuju Chen contributed to the design of the research and the analysis and interpretation of the results. Weidong Lin, Hongxi He, Haibo Wu, and Yali Liu contributed to the experiments and analyzed the data. Bin Li, Qianwen Xi, and Hongyuan Gao recruited the patients enrolled in the study. Renfei Cai, Qiuju Chen, and Haiyan Guo performed the genetic and clinical workup and treatment of patients/couples. All the authors participated in the ultimate interpretation of the study data and approved the final manuscript.

## CONFLICT OF INTEREST STATEMENT

The authors declare no conflicts of interest.

## ETHICS STATEMENT

The study was approved by the Shanghai Ninth People's Hospital Ethics Committee (SH9H‐2022‐T328‐1). Written informed consent was obtained from all participants. All animal studies were approved by the Laboratory Animal Ethics Committee of the Ninth People's Hospital Affiliated with Shanghai Jiao Tong University (SH9H‐2023‐A796‐1).

## Supporting information



Supporting Information

Supporting Information

## Data Availability

The raw sequence data reported in this paper have been deposited in the Genome Sequence Archive (GSA: CRA018867 and HRA008546) of National Genomics Data Center and are publicly accessible at: https://ngdc.cncb.ac.cn/gsa. The other data are available in the Science Data Bank (https://doi.org/10.57760/sciencedb.15339).
