# Supplementary material for: Altered zygotic gene expression caused by sperm with Tdrd6 variants disrupts early embryonic development
Source: MedComm (2020). 2025 Jan 5;6(1):e70038. doi: 10.1002/mco2.70038 (PMC11702384; doi:10.1002/mco2.70038)
Supplement: Supplementary file 1 — Supporting Information [file MCO2-6-e70038-s002.docx]

**Supplementary Information**

**For**

**Altered zygotic gene expression caused by sperm with *Tdrd6* variants disrupts early embryonic development**

Zhijie Hu^1,#^, Yuqi Zhang^1,#^, Renfei Cai^1,#^, Qiuju Chen^1,#^, Haiyan Guo^1,#^, Danjun Li^1,#^, Weidong Lin^2^, Hongxi He^1^, Haibo Wu^1^, Yali Liu^1^, Bin Li^1^, Qianwen Xi^1^, Hongyuan Gao^1^, Jian Zhang^2,*^, Qifeng Lyu^1,*^, Yanping Kuang^1,*^, Xuefeng Lu^1,*^

^1^ Department of Assisted Reproduction, Shanghai Ninth People's Hospital, Shanghai Jiao Tong University School of Medicine, Shanghai 200011, China.

^2^ Medicinal Chemistry and Bioinformatics Center, Shanghai Jiao Tong University School of Medicine, Shanghai 200025, China.

**Supplementary Methods**

**WES and genetic analysis**

Genomic DNA was extracted from peripheral blood samples collected from patients and their family members via a QIAamp DNA Blood Maxi Kit (Qiagen, Dusseldorf, Germany). Exome capture and next-generation sequencing were performed via a TruSeqTM Exome Enrichment Kit (Illumina, San Diego, USA) and an Illumina HiSeq 2500 instrument, respectively. The *TDRD6* variant was confirmed by Sanger sequencing. The variant site in *TDRD6* was amplified by polymerase chain reaction (PCR) using Taq Plus DNA polymerase (Sangon Biotech, Shanghai, China), after which the PCR products were sequenced on a 3730XL sequencer (Applied Biosystems, Foster City, CA, USA) according to the manufacturer’s instructions. The primers used for PCR are listed in Table S4.

**Immunofluorescence staining**

For immunofluorescence (IF) staining of sperm, semen smears fixed in 4% PFA were permeabilized with 1% Triton X-100 in phosphate buffered saline (PBS) for 30 minutes at room temperature (RT), blocked with 5% bovine serum albumin (BSA) for 1 hour, and then incubated with an anti-PLCZ1 polyclonal antibody (1:100 in 5% BSA) (bs-5378R, Bioss, China) or with nonspecific rabbit IgG (HA722127, HUABIO, China) as a negative control at 4°C overnight. The slides were washed with PBS and incubated with Alexa Fluor 488-labeled secondary antibody (1:100, bs-0295G-AF488, Bioss, China) at RT for 1 hour. DNA was stained with 4,6-diamidino-2-phenylindole (DAPI) (H-1200, Vector Laboratories). The slides were observed under a laser scanning confocal microscope (Olympus).

**TEM**

For CB examination, seminiferous tubules were dissected from mouse testes. For sperm morphological observation, semen was washed twice with modified human tubal fluid (mHTF) (90126, Fujifilm Irvine Scientific) medium supplemented with 10% serum substitute supplement (SSS) (99193, Fujifilm Irvine Scientific). The seminiferous tubules and washed sperm pellets were fixed in 2.5% glutaraldehyde (Sigma‒Aldrich) for 24 hours. The sperm samples were washed with phosphate buffer and subsequently fixed in 1% osmium tetroxide for 2 hours. The samples were then dehydrated in a graded series of ethanol and embedded in Epon 618. Ultrathin sections were stained with lead citrate and observed by TEM (HITACHI H-7650, Tokyo, Japan).

**Histological analysis**

The testis and epididymis were fixed in Bouin's solution (G1121, Servicebio, China) for more than 24 hours, dehydrated in a graded series of ethanol, embedded in paraffin and sectioned. Semen smears were prepared and fixed in 4% paraformaldehyde (PFA) (BL539A, Biosharp, China). The sections and smears were stained with hematoxylin and eosin (H&E) according to a standard protocol (G1003, Servicebio, China). Images were captured and scanned by a digital tissue section scanner (Pannoramic MIDI, 3DHISTECH).

**Construction of plasmids for expression and cell transfection**

The full-length coding sequence of *TDRD6* was cloned and inserted into the pcDNA3.1 vector, which includes an HA tag. The full-length coding sequence of *PIWIL1* was cloned and inserted into the p3xFlag-CMV14 vector. *TDRD6* variants were generated via a KOD-Plus Mutagenesis Kit (Toyobo Life Science) according to the manufacturer’s protocol. HEK-293T cells were cultured in Dulbecco's modified Eagle’s medium (DMEM) supplemented with 10% fetal bovine serum (FBS) and 1% penicillin/streptomycin at 37°C in a 5% CO_2_ atmosphere. The plasmids were transfected into HEK-293T cells using Lipofectamine 3000 Reagent (L3000001, Invitrogen) for protein expression.

**Immunoblotting and coimmunoprecipitation (Co-IP)**

Cells were lysed in RIPA buffer (P0013B, Beyotime) supplemented with 1 mM phenylmethanesulfonylfluoride fluoride (PMSF) (ST507, Beyotime) and protease inhibitor cocktail (Sigma) for 20 minutes on ice. The cell lysates were sonicated and centrifuged at 12,000 g for 15 minutes. Loading buffer was added to the protein samples, which were then heated at 100°C for 10 minutes. For immunoblotting, total protein was separated by SDS-PAGE and incubated with specific primary antibodies at 4 °C overnight and then with an HRP-conjugated goat anti-mouse IgG secondary antibody (L3032, Signalway Antibody). An Immobilon Western Chemiluminescent HRP Substrate Kit (Millipore) was used for detection. Monoclonal anti-HA (H9658, Sigma) and monoclonal anti-Flag M2 (F1804, Sigma) primary antibodies were used for immunoblotting.

The washed sperm pallet was lysed according to the cell lyse protocol. Anti-PLCZ1 antibody (human, ab181816, Abcam), PLCZ1 polyclonal antibody (mouse, PA5-98556, Invitrogen), beta actin antibody (HRP-60008, Proteintech), and GAPDH rabbit polyclonal antibody (LF206, Epizyme) were used for immunoblotting. HRP-labeled goat anti-rabbit IgG (LF102, Epizyme) was used as secondary antibody.

Immunoprecipitation was carried out via an HA-tag protein immunoprecipitation kit (P2185S, Beyotime) according to the manufacturer’s protocol.

**Generation of the mouse model**

The *Tdrd6 ^-/-^* mouse model was generated via CRISPR-Cas9 technology. The guide RNAs (Table S5) and Cas9 mRNA were pooled and injected into C57BL/6 mouse zygotes. For the *Tdrd6^N1015Tfs*3/N1015Tfs*3^* mouse model, the guide RNA (Table S5), the donor oligo containing the c.3041_3042 delinsC variant and synonymous variant (p.A1011, GCG to GCT), and Cas9 mRNA were coinjected into C57BL/6 mouse zygotes to generate targeted knock in offspring. F0 founder animals were identified by PCR followed by sequence analysis and were bred with wild-type C57BL/6 mice to obtain offspring. The primers used for genotype identification are listed in Table S4.

**Mouse oocyte collection**

C57BL/6 female mice (4 to 6 weeks old) were superovulated via the injection of 10 IU pregnant mare serum gonadotropin (PMSG) (P9970, Solarbio) followed by 10 IU human chorionic gonadotropin (hCG) (Ningbo Second Hormone Factory, Zhejiang, China) 48 hours later. Cumulus-oocyte complexes (COCs) were retrieved in modified human tubal fluid medium (mHTF) (90126, Fujifilm Irvine Scientific) medium supplemented with 10% serum substitute supplement (SSS) (99193, Fujifilm Irvine Scientific) 14-16 hours after hCG injection. The metaphase II (MII) oocytes were washed several times with hyaluronidase (H4272, Sigma-Aldrich) (70 mg/ml) to remove cumulus cells.

**Embryo transfer in mice**

Female ICR mice (8 to 12 weeks old) were used as pseudopregnant mothers and were mated with vasectomized ICR male mice the night before embryo transfer. Suitable surrogate ICR mice were identified on the basis of the presence of a vaginal plug. Two-cell zygotes were transferred into the recipient's oviducts via a glass pipette. Pups were born through natural labor.

**Ca^2+^ monitoring**

Rhod-2 (R1245MP, Invitrogen), a calcium indicator with fluorescence excitation and emission maxima at 552 nm and 581 nm, respectively, was dissolved in DMSO (D2650, Sigma) to a concentration of 1 mM. MII oocytes were stained with Rhod-2 dissolved in mHTF at a working concentration of 1 µM and incubated in the dark at 37 °C for 20 minutes. After the sperm head was injected into the oocytes, the oocytes were washed three times in mHTF and then placed in a drop of mHTF under mineral oil equipped with an atmospheric chamber. Scans were performed every 5 seconds over a period of 30 minutes. The Ca^2+^ signal was quantified by averaging the signal collected over the whole oocyte area.

**Real-time quantitative PCR (RT‒qPCR)**

Each single-embryo transcriptome was amplified via a single-cell sequence-specific amplification kit (P621, Vazyme Biotech, Nanjing, China). Real-time quantitative PCR was performed with TB Green Premix Ex Taq (R420Q, Takara, Japan) on a QuantStudio 6-Flex real-time PCR system (Applied Biosystems, Life Technologies) according to the manufacturer’s instructions. *Actin* was used as an internal control. All primers used for RT‒qPCR are listed in Table S6

***Mos* transcript degradation test**

To prepare mRNAs for microinjection, GFP was fused with an untailed *Mos* 3'-UTR, mCherry was fused with an untailed *Tubb3* 3'-UTR (unaffected gene used as an internal control), and BFP (coding sequence) was fused with a polyA tail (normalization control). In vitro transcription of GFP-*Mos* 3'-UTR, mCherry-*Tubb3* 3'-UTR and BFP-polyA was performed with a HiScribe T7 ARCA mRNA Kit (E2060S, New England Biolabs) according to the manufacturer’s protocol. A MEGAclear Transcription Clean-Up Kit (AM1908, Invitrogen) was used for mRNA purification. The synthesized RNAs were stored at −80°C until use. GFP-*Mos* 3'-UTR, mCherry-*Tubb3* 3'-UTR and BFP-polyA (300 ng/µl in RNase-free water) were coinjected into MII oocytes. These oocytes were then subjected to ICSI using WT or *Tdrd6^-/-^* sperm. After 6 hours, the zygotes (at the PN4 stage) were scanned under a laser scanning confocal microscope (Olympus). The fluorescence intensity was calculated with ImageJ.JS.

**ROS detection**

After the MII oocytes were obtained, they were cultured in KSOM medium or mHTF+10%SSS medium supplemented with 25µg/ml vitamin C (A0278, Sigma-Aldrich). The oocytes were subjected to ICSI or ICSI-AOA with different concentrations of ionomycin using WT or *Tdrd6 ^-/-^* sperm. The ROS in these oocytes were subsequently detected via an intracellular ROS fluorescence Assay (MAK145, Sigma-Aldrich). These oocytes were scanned under a laser scanning confocal microscope (Olympus). The fluorescence intensity was calculated with ImageJ.JS.


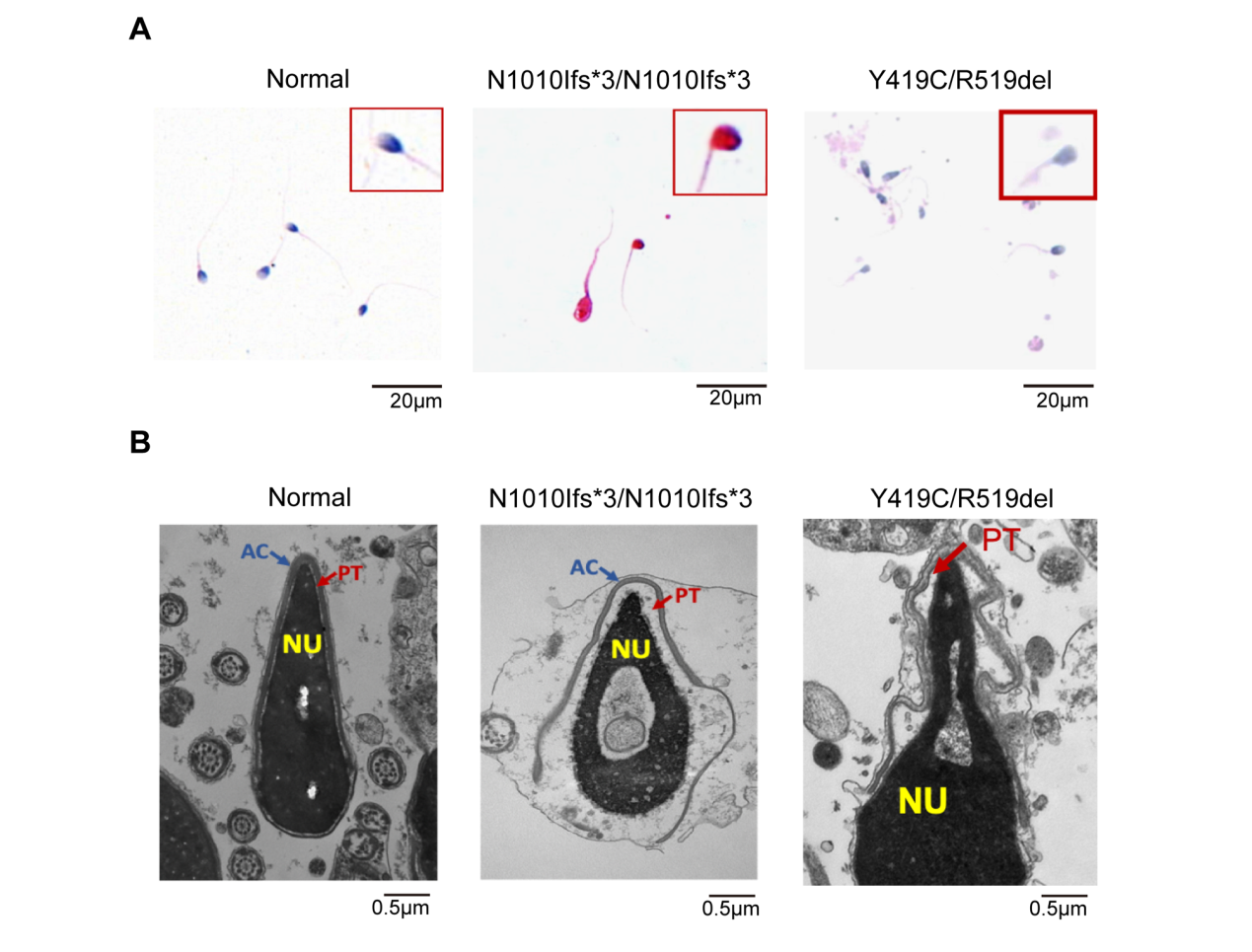


**FIGURE S1.** Sperm morphology in affected individuals. (A) HE staining of sperm from normal donors and patients with *TDRD6* variants. The panel indicated by the red box shows a magnified view of the sperm heads. Scale bars, 20 μm. (B) Transmission electron microscopy images of the ultrastructures of sperm from normal donors and patients with *TDRD6* variants. AC, acrosome; PT, perinuclear theca; and NU, nucleus. Scale bars, 0.5 μm.


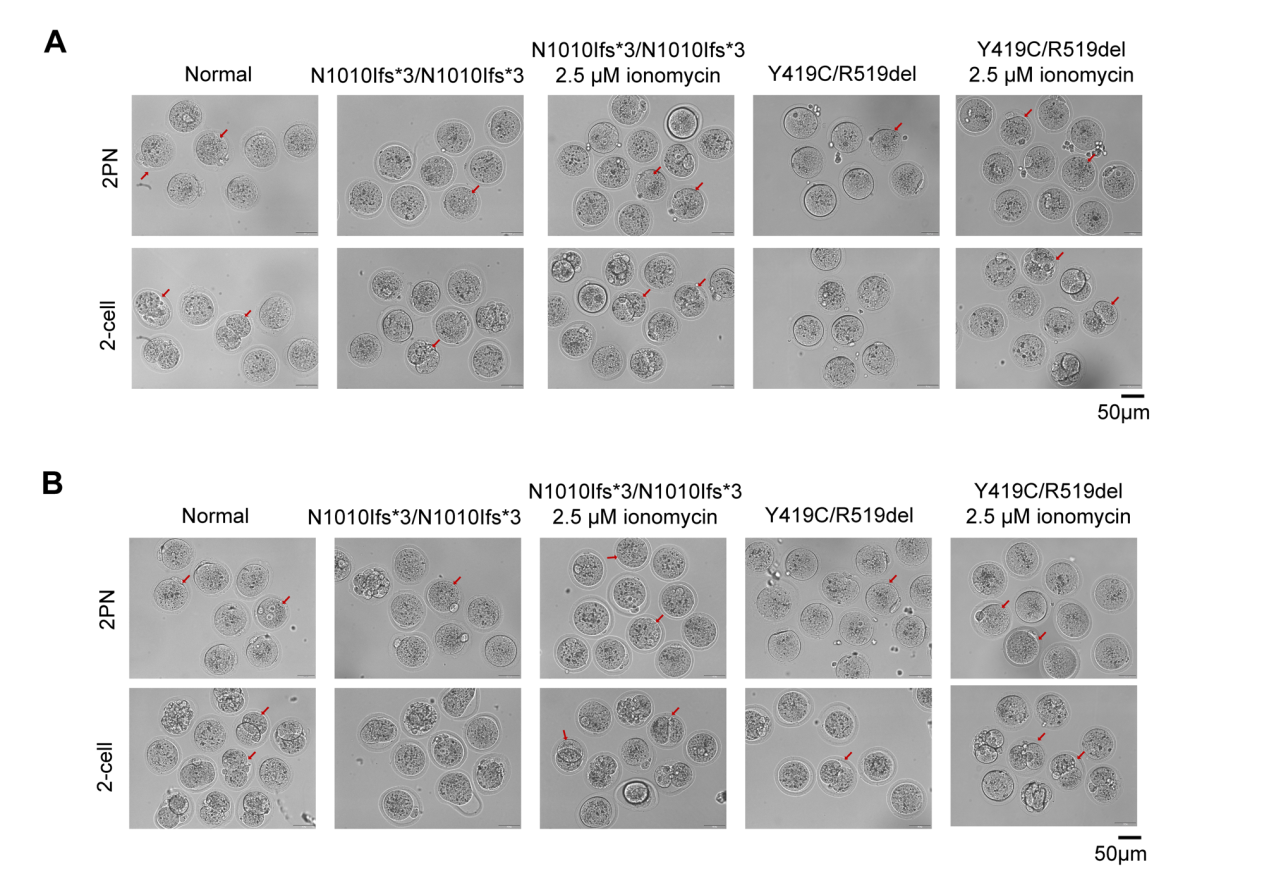


**FIGURE S2.** Images of biological replicates for the mouse oocyte activation test (MOAT). (A) Representative images of 2PN zygotes and 2-cell embryos generated via ICSI or ICSI-AOA of WT mouse oocytes using sperm from normal donors or patients with *TDRD6* variants for the second repeat. Scale bars, 50 μm. (B) Representative images of 2PN zygotes and 2-cell embryos generated via ICSI or ICSI-AOA of WT mouse oocytes using sperm from normal donors or patients with *TDRD6* variants for the third repeat. Scale bars, 50 μm.


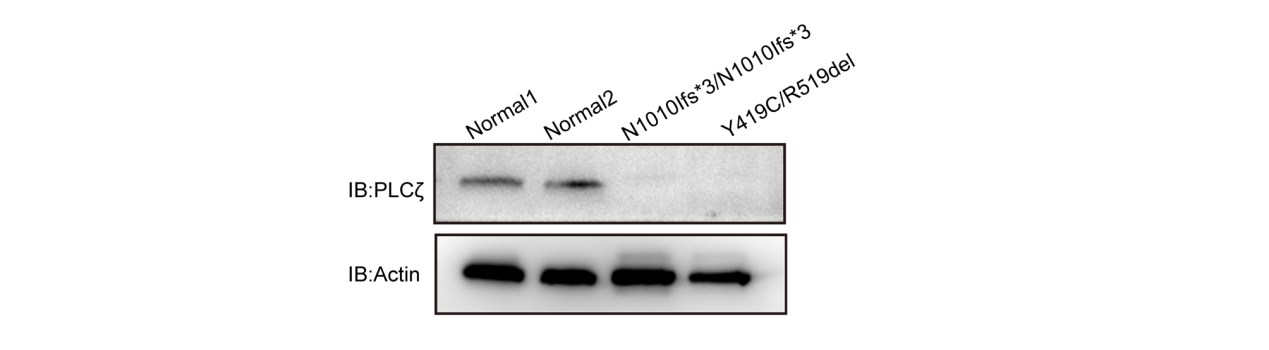


**FIGURE S3.** The expression of PLCζ in the sperm from patients with *TDRD6* variants and normal donors. β-Actin served as a loading control.


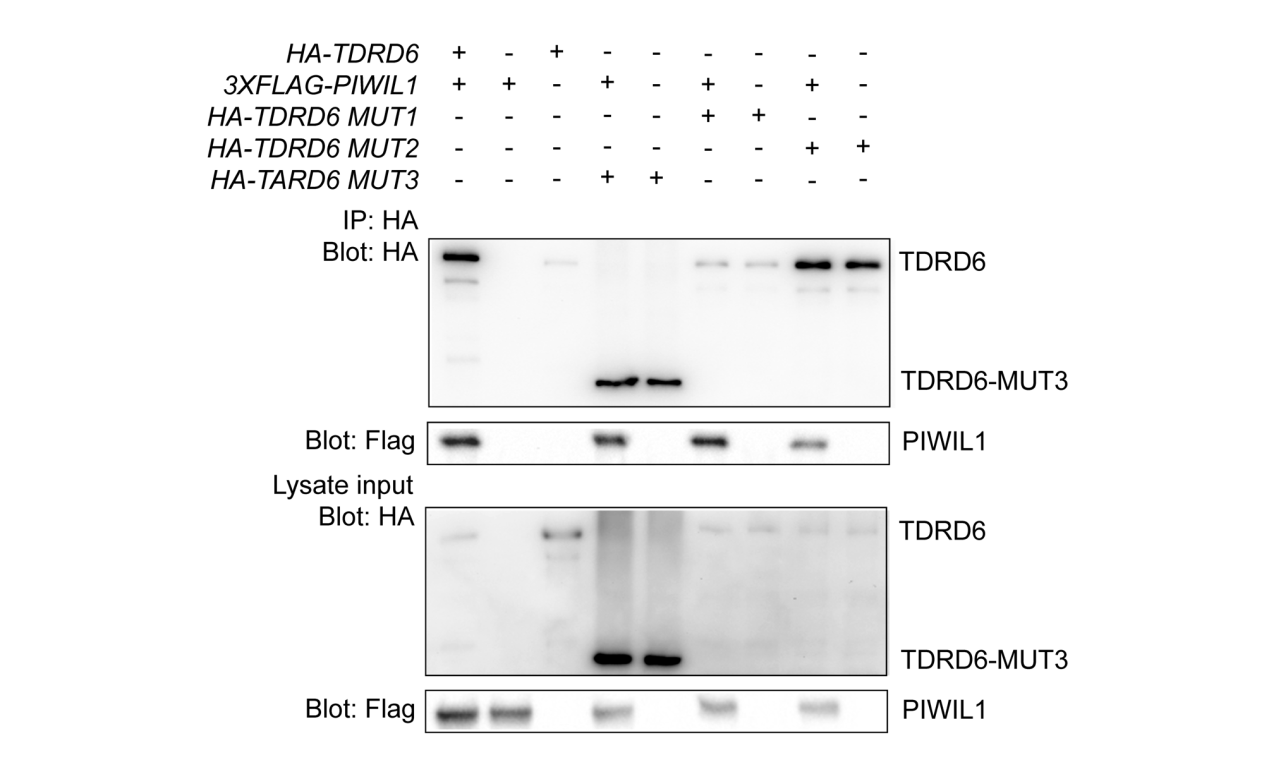


**FIGURE S4.** Coimmunoprecipitation of PIWIL1 with TDRD6. *HA-TDRD6 MUT1*: c.A1256G; *HA-TDRD6 MUT2*: c.1550-1553delinsT; *HA-TDRD6 MUT3*: c.3026-3027delinsC.


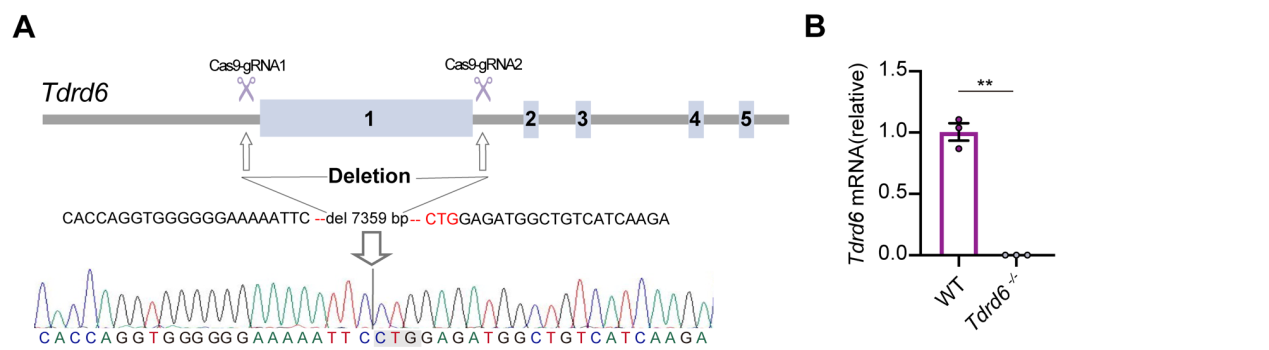


**FIGURE S5.** Generation of *Tdrd6* knock-out mice. (A) Schematic diagram of the *Tdrd6* knock-out strategy. (B) qRT‒PCR was used to measure the relative expression of *Tdrd6* in the testes of WT and *Tdrd6 ^-/-^* male mice. The bars indicate the means ± SEMs. n = 3 biologically independent male mice in each group. ^*^P < 0.05, ^**^P < 0.01, and ^***^P < 0.001.


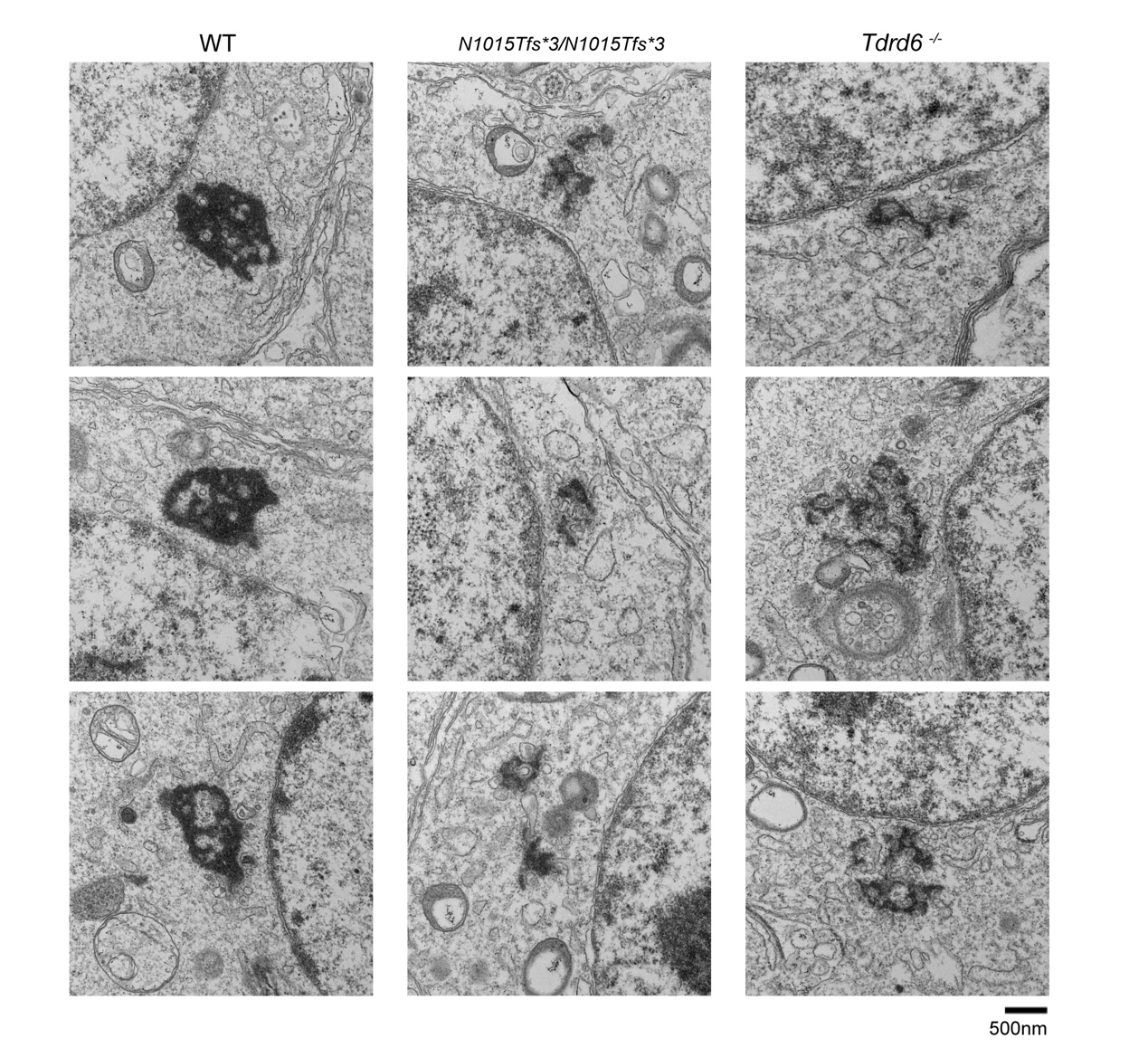


**FIGURE S6.** Transmission electron microscopy image of the chromatiod bodies in round spermatids in WT, *Tdrd6^N1015Tfs*3/N1015Tfs*3^* and *Tdrd6 ^-/-^* male mice. Scale bars, 500nm.

**
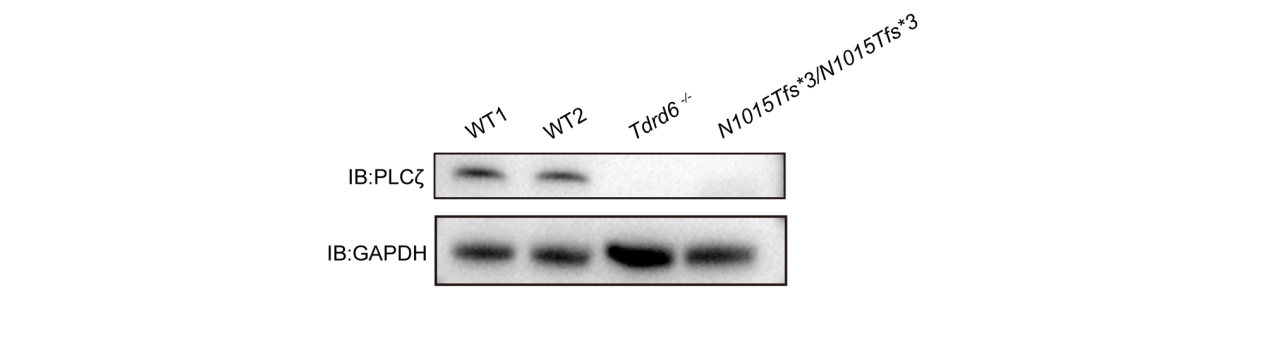
**

**FIGURE S7.** The expression of PLCζ in the sperm of WT, *Tdrd6^N1015Tfs*3/N1015Tfs*3^* and *Tdrd6 ^-/-^* male mice. GAPDH served as a loading control.


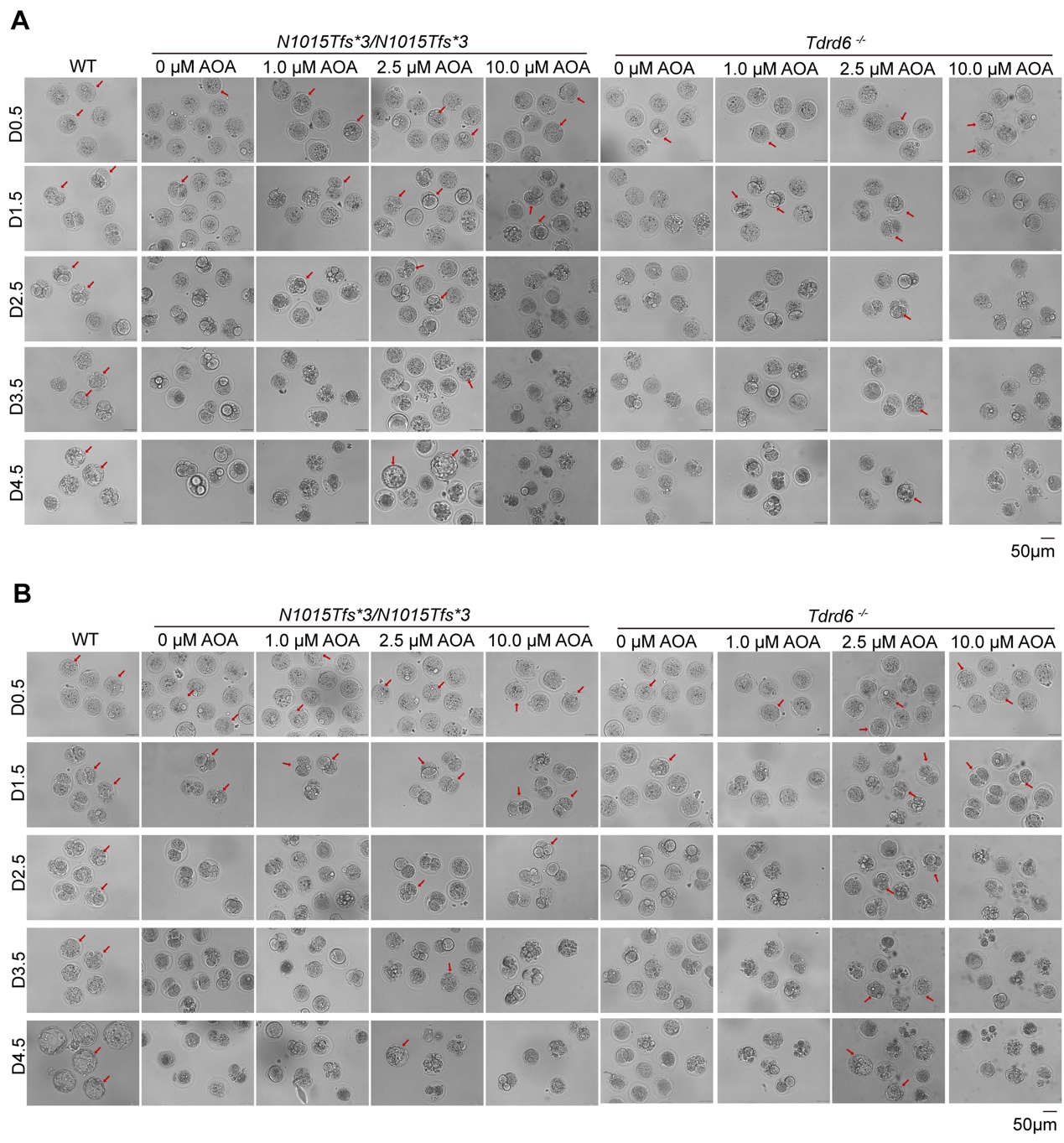


**FIGURE S8.** Images of biological replicates for the embryonic development after ICSI or ICSI-AOA with different concentrations of ionomycin using sperm from WT, *Tdrd6^N1015Tfs*3/N1015Tfs*3^* and *Tdrd6 ^-/-^* mice. (A) Representative images of embryonic development after ICSI or ICSI-AOA with different concentrations of ionomycin using sperm from WT, *Tdrd6^N1015Tfs*3/N1015Tfs*3^* and *Tdrd6 ^-/-^* mice for the second repeat. Scale bars, 50 μm. (B) Representative images of embryonic development after ICSI or ICSI-AOA with different concentrations of ionomycin using sperm from WT, *Tdrd6^N1015Tfs*3/N1015Tfs*3^* and *Tdrd6 ^-/-^* mice for the third repeat. Scale bars, 50 μm. The red arrows indicate the representative 2PN zygotes on D0.5, 2-cell embryos on D1.5, 4-cell embryos on D2.5, morulas on D3.5 and blastocysts on D4.5 respectively.


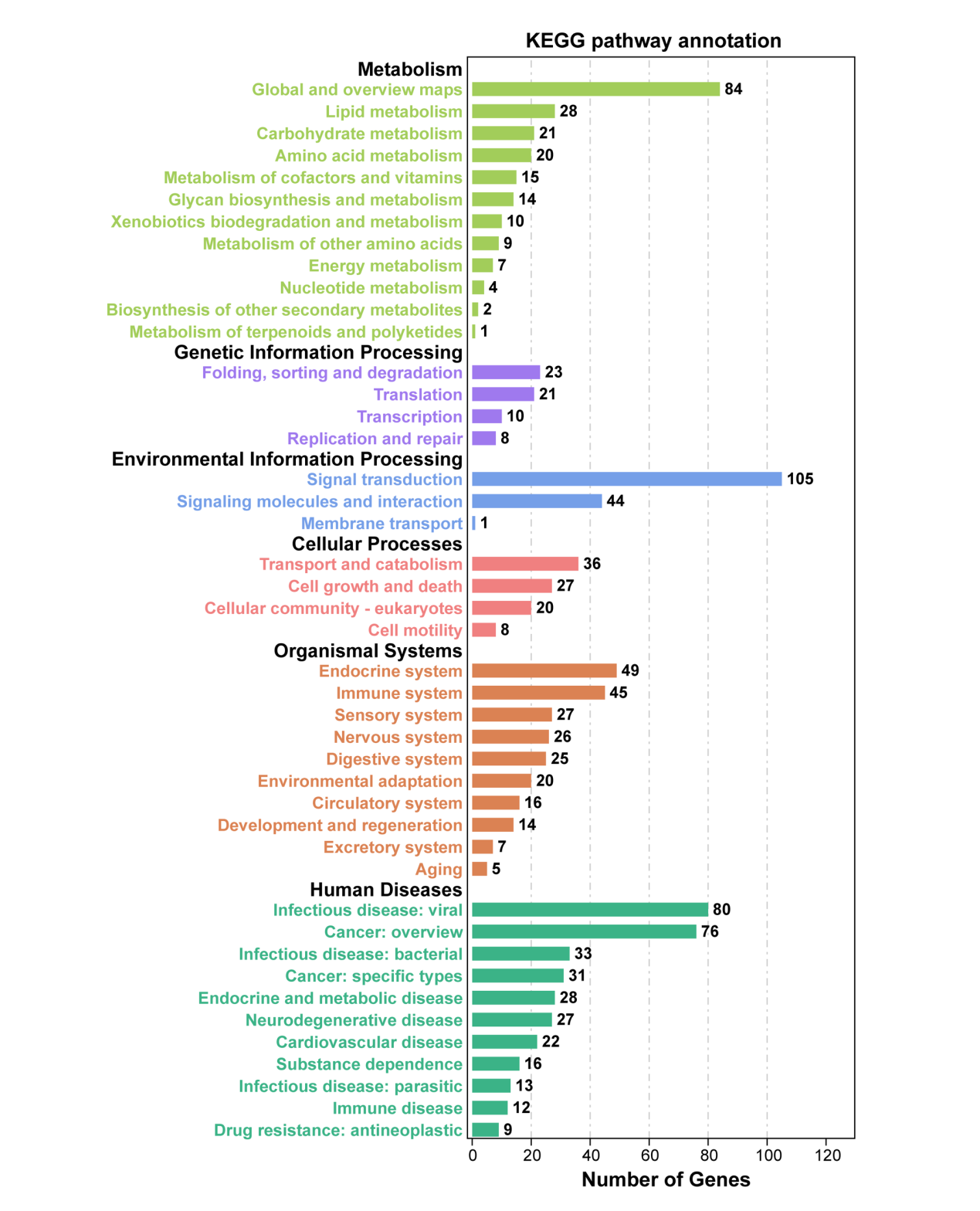


**FIGURE S9.** KEGG pathway analysis of the genes with significant differences in expression observed between the WT group and *Tdrd6 ^-/-^* group and between the *Tdrd6 ^-/-^* group and *Tdrd6 ^-/-^*-ICSI-AOA group, and no significant differences in expression between the WT group and the *Tdrd6 ^-/-^*-ICSI-AOA group.


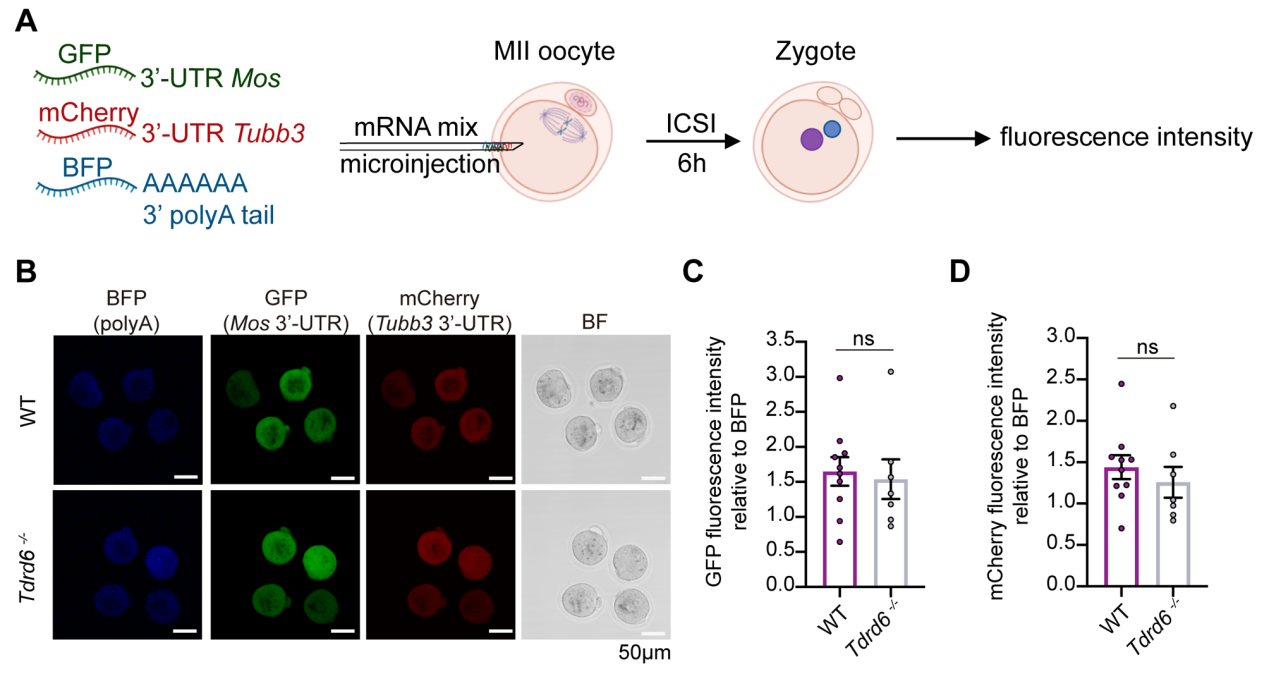


**FIGURE S10.** Degradation of the *Mos* transcript through its 3’-UTR in zygotes generated via ICSI using WT or *Tdrd6 ^-/-^* sperm. (A) The schematic diagram of mRNA microinjection and ICSI used to investigate whether *Tdrd6 ^-/-^* sperm influence the degradation of *Mos* transcript. The GFP transcript was fused with the *Mos* 3’-UTR, mCherry was fused with the *Tubb3* 3’-UTR (an unaffected gene as an internal control), and BFP was fused with polyA (normalization control). (B) Representative fluorescence images of zygotes 6 hours after mRNA microinjection and ICSI with WT or *Tdrd6 ^-/-^* sperm. Scale bars, 50 μm. (C) Relative GFP fluorescence intensity of zygotes in the WT group and *Tdrd6 ^-/-^* group. The bars indicate the means ± SEMs. (D) Relative mCherry fluorescence intensity of zygotes in the WT group and *Tdrd6 ^-/-^* group. The bars indicate the means ± SEMs.


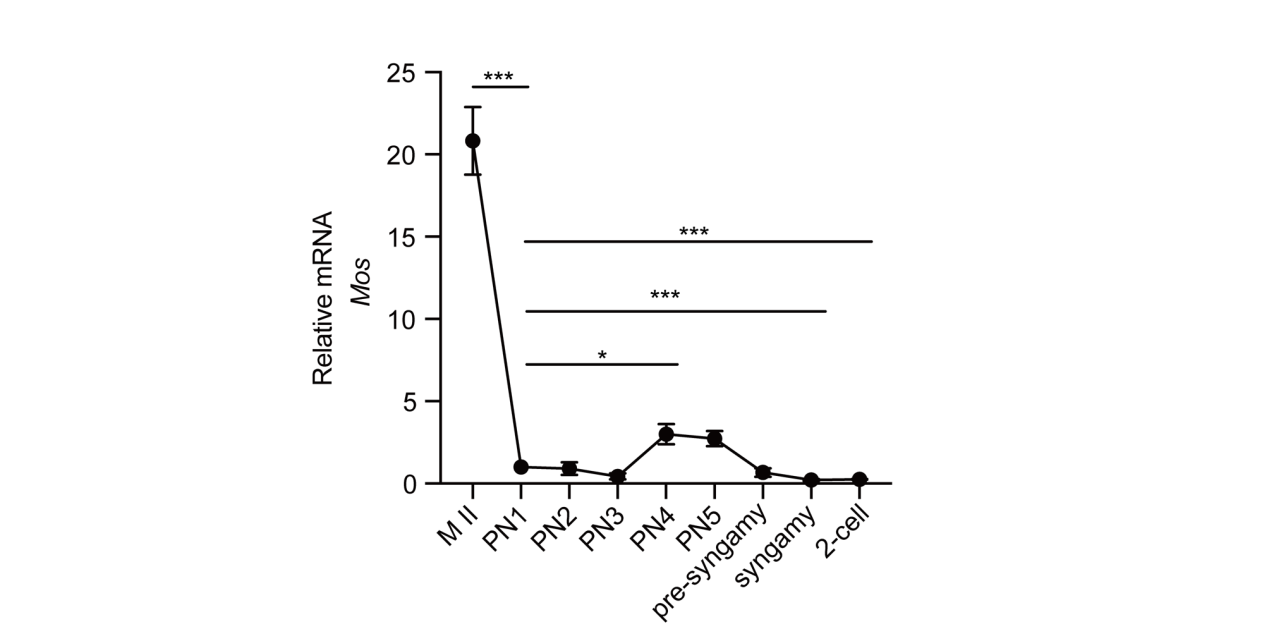


**FIGURE S11.** *Mos* mRNA expression in WT MII oocytes, zygotes and 2-cell embryos. The bars indicate the means ± SEMs. ^*^P < 0.05, ^**^P < 0.01, and ^***^P < 0.001.

**
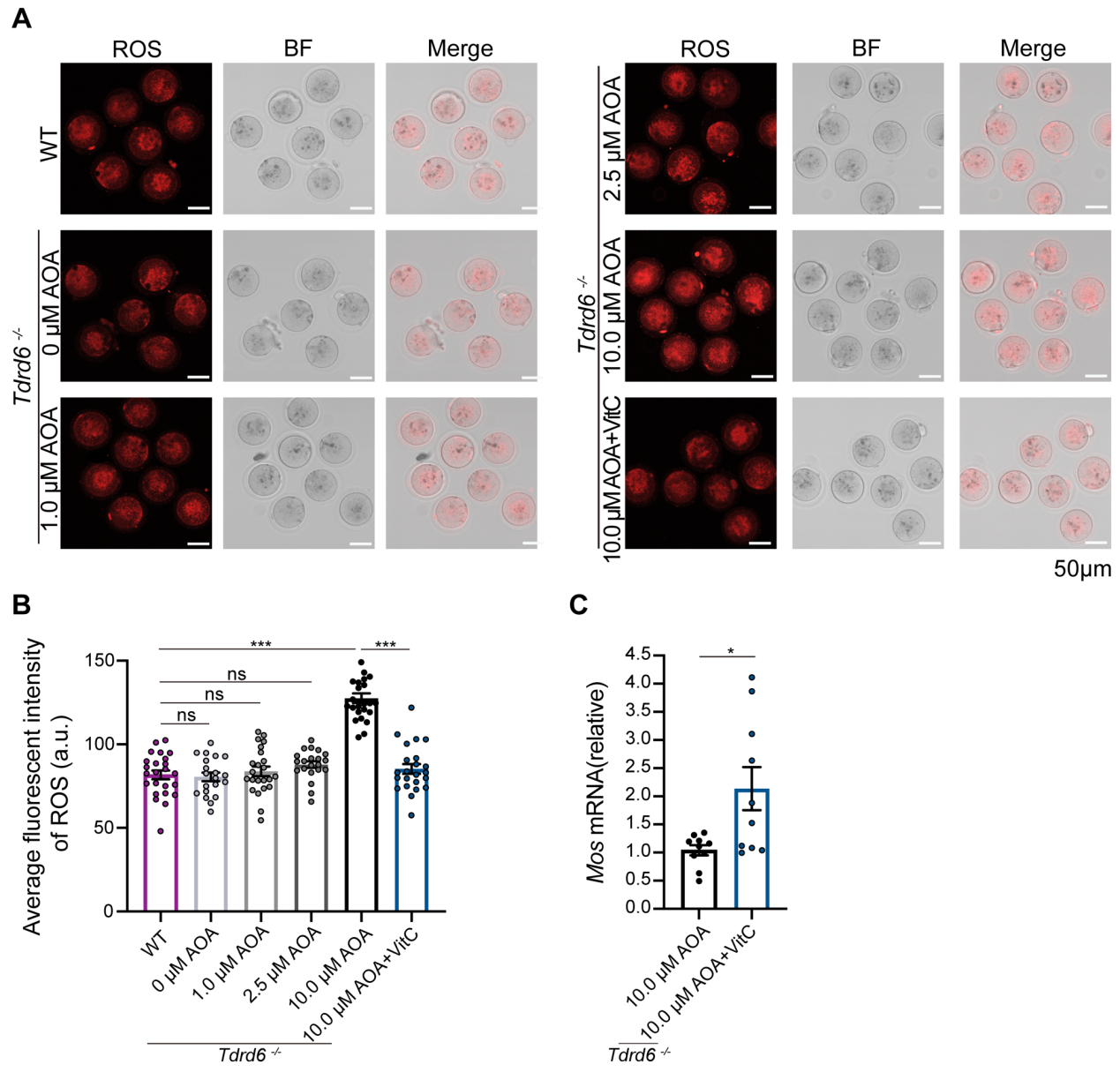
**

**FIGURE S12.** Reactive oxygen species (ROS) of the oocytes after ICSI or ICSI-AOA with different concentrations of ionomycin using WT or *Tdrd6 ^-/-^* sperm. (A) Representative ROS fluorescence images of oocytes after ICSI or ICSI-AOA with different concentrations of ionomycin using WT or *Tdrd6 ^-/-^* sperm. Scale bars, 50 μm. (B) ROS fluorescence intensity of oocytes after ICSI or ICSI-AOA with different concentrations of ionomycin using WT or *Tdrd6 ^-/-^* sperm. The bars indicate the means ± SEMs. n = 3 independent biologically replicates in each group. ^*^P < 0.05, ^**^P < 0.01, and ^***^P < 0.001. (C) qRT‒PCR was used to measure the relative expression of *Mos* in the oocytes of *Tdrd6 ^-/-^*-10μM AOA group and *Tdrd6 ^-/-^*-10μM AOA+VitC group. The bars indicate the means ± SEMs. n = 3 independent biologically replicates in each group. ^*^P < 0.05, ^**^P < 0.01, and ^***^P < 0.001.

**Table S1.** Bioinformatic analysis of the *TDRD6* variants.

|  | Family 1 II-1 | Family 2 II -1 | Family 2 II -1 |
| --- | --- | --- | --- |
| cDNA | c.3026_3027delinsC | c.A1256G | c.1550_1553delinsT |
| Protein | p.N1010Ifs*3 | p.Y419C | p.R519del |
| Mutation type | frameshift substitution | nonsynonymous SNV | nonframeshift substitution |
| Polyphen-2^†^ HumDiv | N/A | Probably damaging (1.000) | N/A |
| Polyphen-2 HumVar | N/A | Probably damaging (0.997) | N/A |
| SIFT^‡^ | N/A | Damaging (0.003) | N/A |
| PROVEAN^§^ | N/A | Deleterious  (-5.96) | Deleterious  (-7.18) |
| GnomAD exome^¶^ | N/A | 0.000354 | N/A |
| ExAC^∥^ | N/A | 0.000330 | N/A |
| 1000 Genomes^∗∗^ | N/A | 0.0018 | N/A |

NA, not available. ^†^ Prediction scores range from 0 to 1, with high scores indicating possibly or probably damaging. ^‡^ Scores vary between 0 and 1; variants with scores close to or equal to 0 are predicted to be damaging. ^§^ Variants with scores lower than −2.5 (cutoff) are predicted to be deleterious. ^¶^ Allele variation frequency in the total gnomAD exome database. ^∥^Allele variation frequency in total ExAC database. ^∗∗^ Allele variation frequency in the 1000 Genomes database.

**Table S2.** Semen analysis of affected individuals.

| Semen analysis | Family 1 Ⅱ-1 | Family 2 Ⅱ-1 | Low reference limits^†^ |
| --- | --- | --- | --- |
| Semen volume (mL) | 4.5 | 2 | 1.4 (1.3-1.5) |
| Sperm concentration (10^6^/mL) | <2 | 31.59 | 16 (15.0-18.0) |
| Progressive motility (%) | / | 3.13 | 30 (29.0-31.0) |
| Total motility (%) | B-grade sperm is extremely occasionally visible | 18.76 | 42 (40.0-43.0) |
| Morphologically normal forms (%) | 0 | 0 | 4 (3.9-4.0) |

^†^ Low reference limits according to the standards in the Sixth Edition of the WHO Manual.

**Table S3.** Primers for target gene PCR.

| Primer name | Primer Sequence (5’ to 3’) |
| --- | --- |
| *T7Mos*-F | TAATACGACTCACTATAGGGGGCATGCCTTCGCCTCTAAG  CCTGT |
| *T7Mos*-R | TCAGCCTAGTGCCCCTCGGAAA |
| *T7Camkk2*-F | TAATACGACTCACTATAGGGGGCATGTCATCATGTGTCT  CTAGCCAGC |
| *T7Camkk2*-R | CTACTCCGGCTCCATCACCTC |

**Table S4.** Primers for verification of *TDRD6* variants and mouse genotyping.

| Primer name | Primer Sequence (5’ to 3’) |
| --- | --- |
| *TDRD6*-1F | AGAAGCCTTTGGAGTCCTCTGTTC |
| *TDRD6*-1R | ACTAGGTATTGGAAGCAGATCATC |
| *TDRD6*-2F | TGGACTATGGAAGGAAGGAG |
| *TDRD6*-2R | TTCAGCAGGAGACTGAGAC |
| *TDRD6*-3F | AGGAGGAGGAACCAGAAAC |
| *TDRD6*-3R | AGGAAGCAGCATCCTTACG |
| *Tdrd6 ^-/-^*-1F | GGATCTGTCATTCAGACACGAAAC |
| *Tdrd6 ^-/^*^-^-1R | TCACTGATGCTGTGGGAGGGAC |
| *Tdrd6 ^-/-^*-2R | AGAGGCGATCCTTCGTCCAG |
| *Tdrd6^N1015Tfs*3/ N1015Tfs*3^* -F | CCTTCCGTTCAGCTACACTCTTAC |
| *Tdrd6^N1015Tfs*3/ N1015Tfs*3^* -R | GGATGTCCGATAAGGAGCATTTCA |

**Table S5**. gRNA target sequence.

| gRNA name | Sequence (5’ to 3’) |
| --- | --- |
| *Tdrd6 ^-/-^*- gRNA1 | TTTCTCTTCGAGAACTCCAATGG |
| *Tdrd6 ^-/-^*- gRNA2 | TCCAGTGGGAGTAATGGCTATGG |
| *Tdrd6^N1015Tfs*3/ N1015Tfs*3^* - gRNA | CGTTTGTATTTCTCGCGAGCTGG |

**Table S6.** Primers for Real-time quantitative PCR.

| Primer name | Primer Sequence (5’ to 3’) |
| --- | --- |
| *Actin*-F | CCTAGGCACCAGGGTGTGAT |
| *Actin*-R | AGCAGGGTGCTCCTCA |
| *Mos*-F | TGGCTGGTTTTGAGAATCAAGG |
| *Mos*-R | GTCACATGAGACACATAGGGAGA |
| *Camkk2*-F | TGCGGGGACTCTCATCCTTA |
| *Camkk2*-R | GGGACATCTTGCGACCAGAA |

**Supplemental material**

Supplemental material for this article is available in the Supplementary file.docx
